# Dual-Site Phosphorylation of the Control of Virulence Regulator Impacts Group A Streptococcal Global Gene Expression and Pathogenesis

**DOI:** 10.1371/journal.ppat.1004088

**Published:** 2014-05-01

**Authors:** Nicola Horstmann, Miguel Saldaña, Pranoti Sahasrabhojane, Hui Yao, Xiaoping Su, Erika Thompson, Antonius Koller, Samuel A. Shelburne

**Affiliations:** 1 Department of Infectious Diseases, MD Anderson Cancer Center, Houston, Texas, United States of America; 2 Department of Bioinformatics and Computational Biology, MD Anderson Cancer Center, Houston, Texas, United States of America; 3 DNA Sequencing Facility, MD Anderson Cancer Center, Houston, Texas, United States of America; 4 Department of Pathology, Stony Brook University Medical Center, Stony Brook, New York, United States of America; 5 Department of Genomic Medicine, MD Anderson Cancer Center, Houston, Texas, United States of America

## Abstract

Phosphorylation relays are a major mechanism by which bacteria alter transcription in response to environmental signals, but understanding of the functional consequences of bacterial response regulator phosphorylation is limited. We sought to characterize how phosphorylation of the control of virulence regulator (CovR) protein from the major human pathogen group A *Streptococcus* (GAS) influences GAS global gene expression and pathogenesis. CovR mainly serves to repress GAS virulence factor-encoding genes and has been shown to homodimerize following phosphorylation on aspartate-53 (D53) *in vitro*. We discovered that CovR is phosphorylated *in vivo* and that such phosphorylation is partially heat-stable, suggesting additional phosphorylation at non-aspartate residues. Using mass spectroscopy along with targeted mutagenesis, we identified threonine-65 (T65) as an additional CovR phosphorylation site under control of the serine/threonine kinase (Stk). Phosphorylation on T65, as mimicked by the recombinant CovR T65E variant, abolished *in vitro* CovR D53 phosphorylation. Similarly, isoallelic GAS strains that were either unable to be phosphorylated at D53 (CovR-D53A) or had functional constitutive phosphorylation at T65 (CovR-T65E) had essentially an identical gene repression profile to each other and to a CovR-inactivated strain. However, the CovR-D53A and CovR-T65E isoallelic strains retained the ability to positively influence gene expression that was abolished in the CovR-inactivated strain. Consistent with these observations, the CovR-D53A and CovR-T65E strains were hypervirulent compared to the CovR-inactivated strain in a mouse model of invasive GAS disease. Surprisingly, an isoalleic strain unable to be phosphorylated at CovR T65 (CovR-T65A) was hypervirulent compared to the wild-type strain, as auto-regulation of *covR* gene expression resulted in lower *covR* gene transcript and CovR protein levels in the CovR-T65A strain. Taken together, these data establish that CovR is phosphorylated *in vivo* and elucidate how the complex interplay between CovR D53 activating phosphorylation, T65 inhibiting phosphorylation, and auto-regulation impacts streptococcal host-pathogen interaction.

## Author Summary

Group A *Streptococcus* (GAS) causes a variety of human diseases ranging from mild throat infections to deadly invasive infections. The capacity of GAS to cause infections at such diverse locations is dependent on its ability to precisely control the production of a broad variety of virulence factors. The control of virulence regulator (CovR) is a master regulator of GAS genes encoding virulence factors. It is known that CovR can be phosphorylated on aspartate-53 *in vitro* and that such phosphorylation increases its regulatory activity, but what additional factors influence CovR-mediated gene expression have not been established. Herein we show for the first time that CovR is phosphorylated *in vivo* and that phosphorylation of CovR on threonine-65 by the threonine/serine kinase Stk prevents aspartate-53 phosphorylation, thereby decreasing CovR regulatory activity. Further, while CovR-mediated gene repression is highly dependent on aspartate-53 phosphorylation, CovR-mediated gene activation proceeds via a phosphorylation-independent mechanism. Modifications in CovR phosphorylation sites significantly affected the expression of GAS virulence factors during infection and markedly altered the ability of GAS to cause disease in mice. These data establish that multiple inter-related pathways converge to influence CovR phosphorylation, thereby providing new insight into the complex regulatory network used by GAS during infection.

## Introduction

Bacteria causing infections in humans must closely modulate virulence factor production in response to different environmental challenges [1], [2], [3]. It has long been recognized that two-component gene regulatory systems (TCS) are a major mechanism by which bacteria react to external stimuli, and thus are critical to the virulence of numerous pathogenic bacteria [4], [5], [6], [7]. Although there is diversity in TCS composition [8], standard TCS consist of a membrane-embedded histidine kinase that can respond to environmental signals by either phosphorylating or dephosphorylating a cognate response regulator, usually on an aspartate residue in the N-terminal receiver domain [9]. The aspartate phosphorylation status of the response regulator alters its gene regulation effect thereby allowing the organism to remodel its expression profile [10].

The critical role of TCS is exemplified by their status as possible targets for novel antimicrobials [11], [12], [13], [14]. However, much remains to be learned about the mechanisms and effects of bacterial response regulator phosphorylation. One of the model systems for understanding how response regulators influence bacterial pathogenesis has been the control of virulence regulator (CovR, also known as CsrR) from group A *Streptococcus* [15], [16], [17]. GAS causes a wide range of infectious and post-infectious syndromes in humans [18]. CovR is a member of the OmpR/PhoB family of response regulators and is a key repressor of numerous GAS virulence factors such that CovR-inactivated strains are hypervirulent [15], [19], [20]. *In vitro* studies have shown that phosphorylation of CovR at amino acid residue aspartate-53 (D53) results in homodimerization and increased DNA-binding affinity, but *in vivo* phosphorylation of CovR has not been demonstrated [21], [22], [23]. *covR* is co-transcribed with an adjacent gene encoding the sensor kinase CovS [15]. Although it has been assumed that CovS controls the D53 phosphorylation status of CovR, numerous groups have reported that inactivation of CovS produces a distinct phenotype from inactivation of CovR suggesting that factors in addition to CovS influence CovR phosphorylation and thus activity [19], [21], [24], [25].

Recently, Agarwal *et al.* found that the GAS eukaryotic-like serine/threonine kinase Stk phosphorylates CovR on threonine residues *in vitro* although neither the exact site nor the functional consequences of such phosphorylation has been determined [26]. GAS Stk and CovR homologues are present in group B *Streptococcus* (GBS), and GBS Stk has been shown to phosphorylate CovR on threonine-65 (T65) [27], [28], [29]. Stk-mediated threonine phosphorylation of GBS CovR resulted in a decreased ability of CovR to be phosphorylated on D53, decreased CovR binding to DNA from the *cylX* promoter, and relieved CovR-mediated repression of the *cyl* operon which encodes the potent β-hemolysin/cytolysin [29], [30], [31]. Whether GAS Stk influences CovR-mediated gene regulation in a fashion similar to GBS is unknown.

To gain new insights into the functional consequence of GAS CovR phosphorylation on host-pathogen interaction, we performed a series of biochemical, genetic, and virulence assays. First, we used the recently developed Phos-Tag technology to demonstrate that CovR is phosphorylated *in vivo*. Using a combination of mass spectrometry, Phos-Tag assays, and targeted mutagenesis, we determined that Stk phosphorylates GAS CovR at T65 and that such phosphorylation negatively influences CovR phosphorylation on D53. In this way, Stk phosphorylation of CovR on T65 significantly impacts GAS global gene expression and virulence. Moreover, we discovered that, in contrast to CovR-mediated gene repression, CovR-mediated activation of gene expression is not primarily determined by CovR D53 phosphorylation status. Taken together, these data provide crucial new information about how the phosphorylation status of the receiver domain of a TCS response regulator influences host-pathogen interaction and provide a key platform for achieving a fuller understanding of the mechanisms influencing response regulator phosphorylation status *in vivo*.

## Results

### CovR is phosphorylated *in vivo*

**Figure 1:**
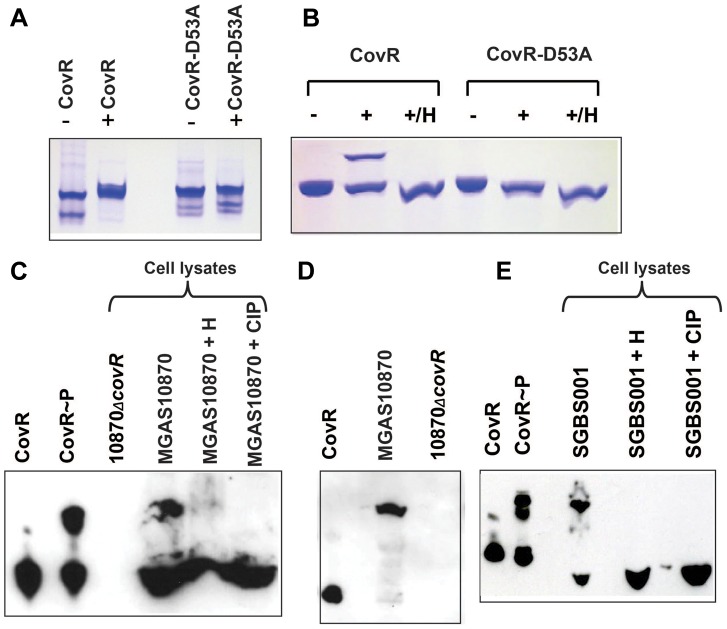
Detection of CovR phosphorylation *in vitro* and *in vivo*. (A and B) Recombinant CovR and CovR-D53A proteins were incubated without (**−**) and with (**+**) acetyl-phosphate. Samples were run on a 12% native polyacrylamide gel (A) or a 12% Phos-tag-SDS polyacrylamide gel (B), respectively. In both cases the higher bands represent the phosphorylated species. The D53A change reduces the negative charge and leads to decreased migration of the protein under native conditions as shown by the higher bands for both lanes in panel (A) compared to unphosphorylated CovR [22]. For (B), samples marked with H were heated for 1 min at 100°C, which removes the heat-labile aspartate phosphorylation. (C) Western blot using anti-CovR antibody of indicated samples separated on a 10% Phos-tag-SDS polyacrylamide gel. CovR∼P indicates recombinant CovR incubated with acetyl-phosphate. Cell lysates were derived from cells grown to mid-exponential phase in THY. H indicates sample was heated whereas CIP indicates treatment with calf intestinal phosphatase. (D) Western blot using anti-CovR antibody as described for (C) except cells were grown in THY supplemented with 15 mM MgCl_2_. (E) Western blot using anti-CovR antibody of cell lysate derived from GBS cells grown to mid-exponential phase in THY and the respective controls as described for (C). For (C–E), cell lysates were loaded at 70 µg of total protein per sample.

Although the importance of CovR phosphorylation has been well established in GAS and GBS over the past 15 years, *in vivo* phosphorylation of CovR has not been demonstrated in either species [22], [27], [28], [29], [30], [32], [33], [34]. Recently, Phos-Tag gels, in which the migration of phosphorylated proteins is retarded compared to non-phosphorylated proteins, have been used to assay protein phosphorylation status *in vitro* and *in vivo* [35], [36], [37], [38]. To investigate whether Phos-Tag gels would be suitable for assessing CovR phosphorylation, we first compared *in vitro* CovR phosphorylation using Phos-Tag gel and native polyacrylamide gel electrophoresis (PAGE), which has been the typical method for studying CovR phosphorylation [22], [39]. Purity of the recombinant proteins used in these assays is shown in Figure S1A in Text S1. Phosphorylation of recombinant CovR by the small molecule phosphodonor acetyl phosphate induces homodimerization, which results in slower migration on a native polyacrylamide gel (Figure 1A) [22]. An alanine substitution at D53 abrogates phosphorylation at the altered site [22] and thereby homodimerization (Figure 1A). Similarly, acetyl-phosphate mediated phosphorylation of wild-type CovR, but not CovR-D53A, could be readily detected using the Phos-Tag method, as demonstrated by the appearance of a higher migrating band in the phosphorylated sample (Figure 1B). Heating of the CovR samples, which removes the heat-labile aspartate-phosphorylation, eliminated the higher running band, confirming that the Phos-tag assay was able to detect CovR aspartate-phosphorylation (Figure 1B) [22].

Next, we sought to use the Phos-Tag assay to determine whether CovR is phosphorylated *in vivo*. To this end, strain MGAS10870, a serotype M3 strain which has been fully sequenced and is known to contain a wild-type CovR/S TCS [40], and its isogenic CovR-inactivated derivative (10870Δ*covR*) were grown to mid-exponential phase in THY medium. The cells were pelleted, lysed, and cell lysates were run on a Phos-Tag gel, transferred to a nitrocellulose membrane, and probed with a polyclonal anti-CovR antibody. The performance of the anti-CovR antibody is shown in Figure S2 in Text S1. For strain MGAS10870, we observed two distinct bands reacting with the anti-CovR antibody which were at the same location as recombinant CovR and recombinant CovR phosphorylated with acetyl phosphate (Figure 1C). These data are consistent with partial CovR phosphorylation in strain MGAS10870 during growth in a standard laboratory medium (THY medium). Heating of the MGAS10870 cell extract led to the disappearance of the majority of the slower migrating band whereas treating of the sample with calf intestinal phosphatase (CIP) completely removed the slower migrating band (Figure 1C). Conversely, no bands were observed in the lysate of strain 10870Δ*covR* (Figure 1C). Next we assayed the phosphorylation status of CovR grown in THY medium supplemented with 15 mM MgCl_2_ as Mg^2+^ is thought to increase CovR D53 phosphorylation [41]. Following growth in a high Mg^2+^ medium, only the slower migrating band was detected by the anti-CovR antibody suggesting that CovR phosphorylation is complete under the high Mg^2+^ condition (Figure 1D). Given that GAS CovR and GBS CovR are highly similar (84% identity, 92% similarity at the amino acid level), we also assayed the cell lysate from the GBS strain SGBS001 and found that our anti-CovR antibody did react with the GBS cell lysate and that GBS CovR appears to be partially phosphorylated during growth in THY (Figure 1E). Phosphorylation of GBS CovR was removed by heat or CIP-treatment. We conclude that the Phos-Tag assays can be used to determine GAS and GBS CovR phosphorylation status *in vitro* and *in vivo* and that during growth in a standard laboratory medium, the majority of GAS CovR phosphorylation is heat-labile, consistent with aspartate phosphorylation.

### Stk phosphorylates CovR on T65 *in vitro*

**Figure 2:**
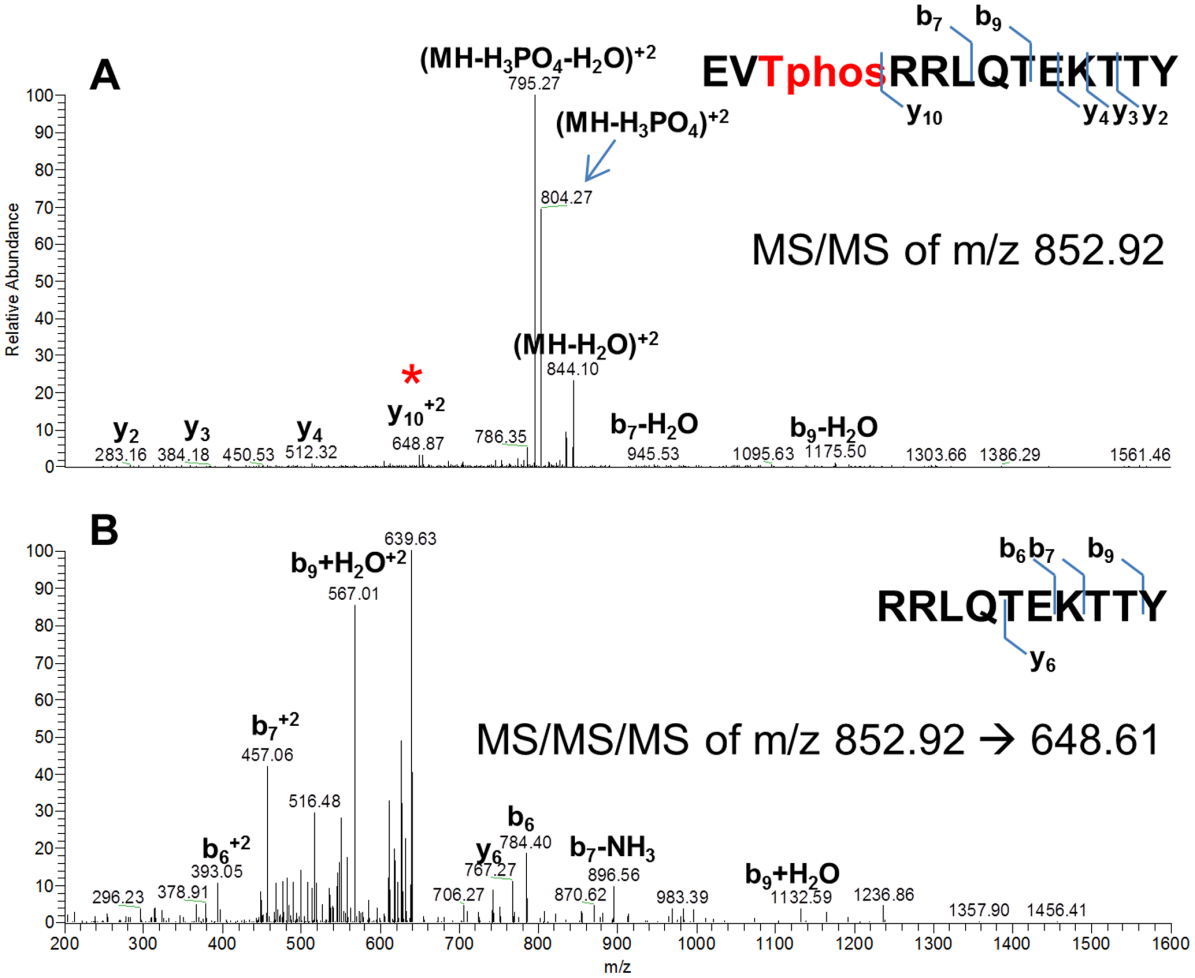
Mass spectra showing that CovR is phosphorylated on threonine 65. Recombinant CovR was incubated with Stk_KD_ and then subjected to chymotrypsin digestion. (A) Mass spectra of the MS/MS fragmentation of a 2^+^ ion of the CovR-derived peptide EVTphosRRLQTEKTTY. Fragment marked with blue arrow shows the peptide that has lost phosphoric acid, thus decreasing the apparent mass from 852.92 to 804.27. The peptide marked with the red star corresponds to the y_10_^2+^ fragment (RRLQTEKTTY) and has not lost phosphoric acid. Thus, T3 must be the position of phosphorylation. (B) Mass spectra of the MS/MS/MS fragmentation of the y_10_^2+^ ion confirmed that the y_10_^2+^ fragment is indeed RRLQTEKTTY. Fragments of corresponding b or y ions are indicated.

The fact that some, but not all, CovR phosphorylation disappeared upon heating suggested that CovR may be phosphorylated at heat-stable sites, such as threonine, in addition to being phosphorylated at the heat-labile aspartate site [35]. It was recently shown that Stk phosphorylates CovR *in vitro* but the phosphorylation site was not identified [26]. Thus, we first sought to determine the amino acid residue in GAS CovR that is the target for Stk mediated phosphorylation. To this end, we overexpressed and purified the kinase domain of Stk (Stk_KD_) from the serotype M3 strain MGAS10870 (Figure S1A in Text S1). Stk_KD_ encompasses the N-terminal 315 amino acids and has been shown previously to be sufficient to phosphorylate CovR on threonine residues *in vitro* [26]. CovR was incubated with Stk_KD_, subjected to chymotrypsin or trypsin digestion, and then analyzed by LC/MS/MS (see Materials and Methods). No specific phosphorylated peptide was detected in the trypsin digest. However, in the chymotrypsin digest, the CovR-derived peptide EVTRRLQTEKTTY was predicted to be phosphorylated at position 3, which corresponds to T65 (Figure 2A). In order to validate the site of phosphorylation we then instructed the mass spectrometer to specifically acquire MS/MS of only this phosphorylated peptide in addition to acquiring MS/MS/MS of specific fragments. Fragmentation of the peptide EVT_65_RRLQTEKTTY showed fragments that lost phosphoric acid (m/z 804, marked with blue arrow in Figure 1A) as well as a specific y_10_^2+^ fragment at m/z 648.61, which was predicted to be RRLQTEKTTY. This fragment was not phosphorylated therefore suggesting that the phosphorylated amino acid residue was T65. Further fragmentation of this y_10_^2+^ fragment proved that this fragment was indeed RRLQTEKTTY (Figure 2B). Thus, *in vitro*, Stk_KD_ phosphorylates CovR on T65.

### Phos-tag gel assays confirm phosphorylation of CovR T65 by Stk *in vitro*

We next sought to confirm our mass spectroscopy finding that T65 is the site of CovR phosphorylation by Stk and to investigate the relationship between CovR D53 and T65 phosphorylation. To this end, we generated a recombinant CovR variant with alanine at the T65 phosphorylation site (CovR-T65A). We performed circular dichroism (CD) spectroscopy on CovR-T65A and the previously generated CovR-D53A (Figure S1A in Text S1) to ensure that the mutations did not result in aberrantly folded proteins. The resulting CD spectra of all recombinant CovR variants were identical to the spectrum of the wild-type CovR protein indicating that our introduced changes had not altered the overall secondary protein structure (Figure S1B in Text S1).

**Figure 3:**
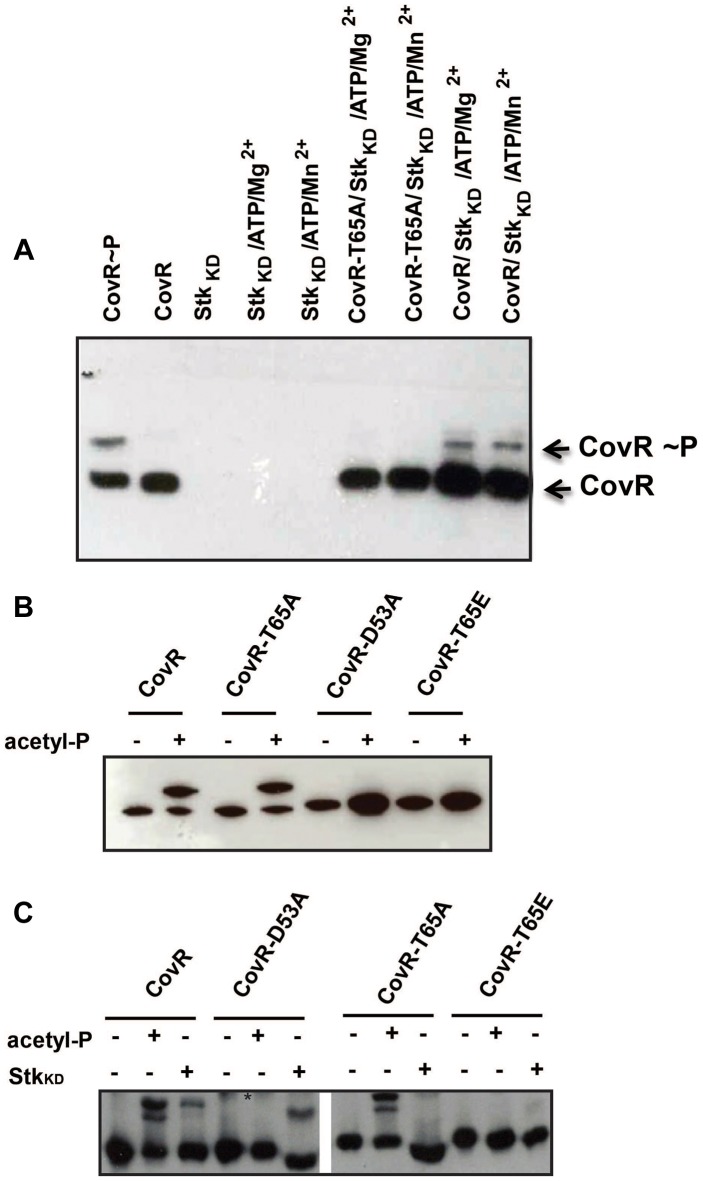
CovR dual-site phosphorylation events influence each other. (A) Western blot using anti-CovR antibody. Samples were separated on a 12% Phos-tag-SDS polyacrylamide gel which was loaded as follows: CovR phosphorylated with acetyl-phosphate as positive control, unphosphorylated CovR as negative control, Stk_KD_ alone, Stk_KD_ in Mg^2+^/ATP buffer or Mn^2+^/ATP buffer; CovR-T65A phosphorylated with Stk_KD_ in Mg^2+^/ATP or Mn^2+^/ATP buffer; CovR phosphorylated with Stk_KD_ in Mg^2+^/ATP or Mn^2+^/ATP buffer. Phosphorylated and unphosphorylated CovR species are labeled. (B) Recombinant wild type and variant CovR proteins were incubated with kinase buffer in the absence (**−**) and presence (**+**) of acetyl-phosphate, separated on a 12% Phos-tag SDS polyacrylamide gel and blotted with anti-CovR antibodies. Note that CovR-D53A and T65E variants cannot be phosphorylated by acetyl-phosphate. (C) Recombinant wild type and variant CovR proteins were incubated with kinase buffer in the absence (**−**) and presence (**+**) of acetyl-phosphate or Stk_KD_, separated on a 12% Phos-tag SDS polyacrylamide gel and blotted with anti-CovR antibody. The retarded bands represent the phosphorylated CovR species. The * denotes unspecific background noted for lanes 4 and 5. Note that the CovR-T65A and T65E variants cannot be phosphorylated by Stk_KD_.

Given that CovR threonine phosphorylation is not expected to induce protein dimerization, native PAGE is not suitable for analysis of CovR threonine phosphorylation status. Thus, we used Phos-tag PAGE to assay for CovR T65 phosphorylation by incubating CovR with Stk_KD_ and then performing standard Western blot using the anti-CovR antibody. This approach eliminates the strong signal from highly phosphorylated (and therefore appearing as several bands) Stk_KD_, which overwhelmed the weaker CovR-P signal when using anti-threonine antibodies (data not shown). As depicted in Figure 3A, the Phos-tag analysis demonstrated wild-type CovR phosphorylation following incubation with Stk_KD_. Although Jin *et al.* found GAS Stk_KD_ to be an Mn^2+^ dependent threonine kinase [42], we did not observe any difference in Stk_KD_-CovR phosphorylation reactions containing 10 mM Mg^2+^ or Mn^2+^ ions, respectively, in the kinase buffer (Figure 3A). Therefore, we used 10 mM MgCl_2_ throughout the following experiments. Consistent with our mass spectrometry data, incubation of CovR-T65A with Stk_KD_ resulted in no detectable phosphorylation using the Phos-tag assay (Figure 3A). In contrast, Stk_KD_ was able to phosphorylate CovR with an alanine substituted for threonine at position 73 (CovR-T73A, data not shown), further supporting the specific role of T65 in Stk-dependent CovR phosphorylation.

### Interplay between acetyl-phosphate and Stk mediated phosphorylation

Having established that Stk phosphorylates CovR on T65, we sought to investigate whether CovR D53 and T65 phosphorylation events influence each other. To this end, we generated the recombinant CovR variant T65E (Figure S1A in Text S1). Introduction of a negative charge at this position mimics constitutive phosphorylation of T65 (i.e. a phospho-mimetic mutation) [29]. Similar to the CovR-D53A and -T65A variants, we observed no difference in CD spectra for the CovR-T65E protein compared to wild-type indicating normal protein folding (Figure S1B in Text S1). Next, we determined whether the CovR variants could be phosphorylated *in vitro* by either acetyl-phosphate or Stk_KD_. By Phos-Tag analysis, CovR-T65A could be phosphorylated by acetyl phosphate similar to wild-type CovR (Figure 3B). However, analogous to the CovR-D53A protein, the T65E substitution in CovR completely impeded acetyl-phosphate mediated phosphorylation (Figure 3B). Consequently, only a phosphor-mimetic (T65E), not a neutral (T65A) mutation at T65 interferes with phosphorylation at CovR D53.

Stk_KD_ was unable to phosphorylate either the CovR-T65A or -T65E variants (Figure 3C). Moreover, Stk_KD_ readily phosphorylated CovR-D53A to the point where we observed reproducibly higher amounts of phosphorylation following incubation with Stk_KD_ for the CovR-D53A protein compared to wild-type CovR (Figure 3C). Hence, *in vitro*, the D53 and T65 CovR phosphorylation sites influence each other.

### Creation and characterization of GAS strains with altered CovR phosphorylation sites

**Figure 4:**
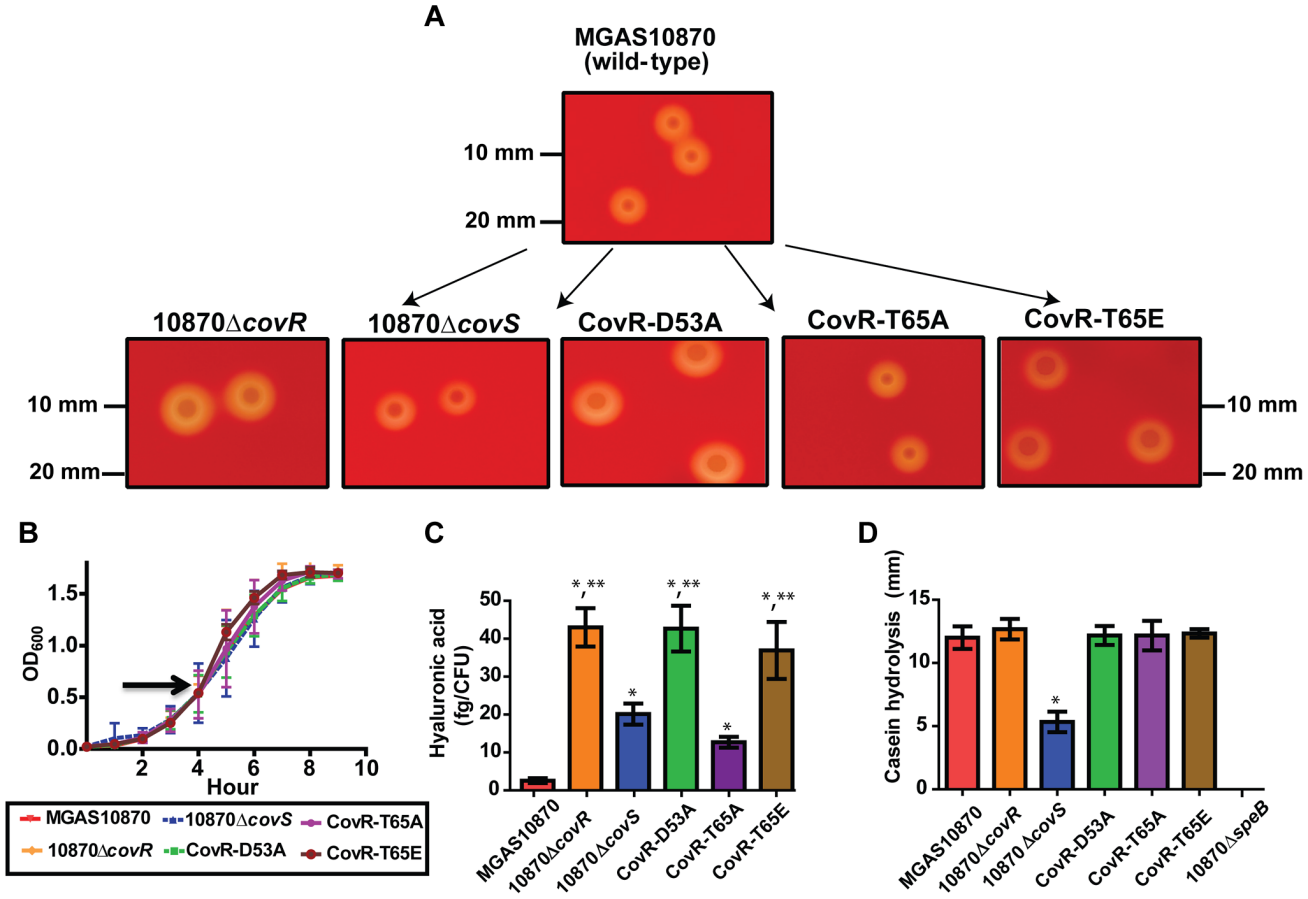
Growth curves and general characterization of GAS CovR/S variants. Various GAS strains were created as described in Materials and Methods. (A) Photographs of indicated strains following overnight growth on 5% sheep blood agar plates. (B) Growth curves of various strains in THY. Arrow shows point where samples were obtained for RNA-Seq analysis. (C) Production of hyaluronic acid capsule following growth in THY. Significant elevation in hyaluronic acid production is indicated by * in comparison to strain MGAS10870 and by ** in comparison to strains MGAS10870, 10870Δc*ovS*, and CovR-T65A as measured by ANOVA followed by Bonferroni's post-hoc test. (D) Casein hydrolysis (marker of SpeB) activity. * indicates significant decrease in casein hydrolysis in strain 10870Δ*covS* compared to the wild-type and CovR variant strains as measured by ANOVA followed by Bonferoni's post-hoc test. Strain 10870Δ*speB* is included as a negative control. For (B–D), strains were grown in triplicate on three separate occasions. Data graphed are mean ± standard deviation.

To better understand the functional consequences of CovR phosphorylation, we next created the following GAS strains from the serotype M3 strain MGAS10870: a CovS-knockout strain (10870Δ*covS*), a strain with an alanine at CovR position 53 (CovR-D53A), a strain with an alanine at CovR position 65 (CovR-T65A), and a strain with glutamate at CovR position 65 (CovR-T65E) (Figure 4A). We previously had created the CovR knockout strain 10870Δ*covR* [43]. The growth curves of the strains in a standard laboratory medium (THY) were indistinguishable (Figure 4B), whereas the phenotypes of the various strains on sheep blood-agar plates were quite distinct (Figure 4A). Compared to wild-type, the 10870Δ*covR*, CovR-D53A, and CovR-T65E strains had a larger colony phenotype indicating increased production of the GAS hyaluronic acid capsule which is known to be negatively regulated by CovR [15]. Conversely, strains 10870Δ*covS* and CovR-T65A had a phenotype similar to strain MGAS10870. Consistent with these observations, strains 10870Δ*covR*, CovR-D53A, and CovR-T65E produced significantly more capsule during growth in THY compared to strains MGAS10870, 10870Δ*covS*, and CovR-T65A (Figure 4C). In many GAS strains, CovS positively influences the production of the cysteine proteinase, streptococcal pyrogenic exotoxin B (SpeB) [44]. Similarly, strain 10870Δ*covS* had significantly decreased SpeB production compared to wild-type as measured by casein hydrolysis (Figure 4D). No significant difference in SpeB production was observed between the CovR variant strains and strain MGAS10870 (Figure 4D).

### Genome-wide characterization of the effects of CovR phosphorylation on GAS gene expression

To determine the genome-wide effects of alterations in CovR phosphorylation status on GAS gene expression, four replicates of each strain were grown to mid-exponential phase (Figure 4B), and transcriptome analyses were performed using RNA-Seq. A minimum of 2.3 million paired reads were resolved per strain for a minimum average base coverage of 174 indicating excellent sequencing depth. Gene transcripts were detected for 1609 genes or approximately 83% of the 1951 genes in strain MGAS10870. Principal components analysis, which creates a global view of the variance in the data set, showed that strains 10870Δ*covR*, CovR-D53A, and CovR-T65E were the most distant from wild-type (Figure S3 in Text S1). Strain 10870Δ*covS* was closest to wild-type whereas strain CovR-T65A had a phenotype intermediate between wild-type and strain 10870Δ*covR*.

**Figure 5:**
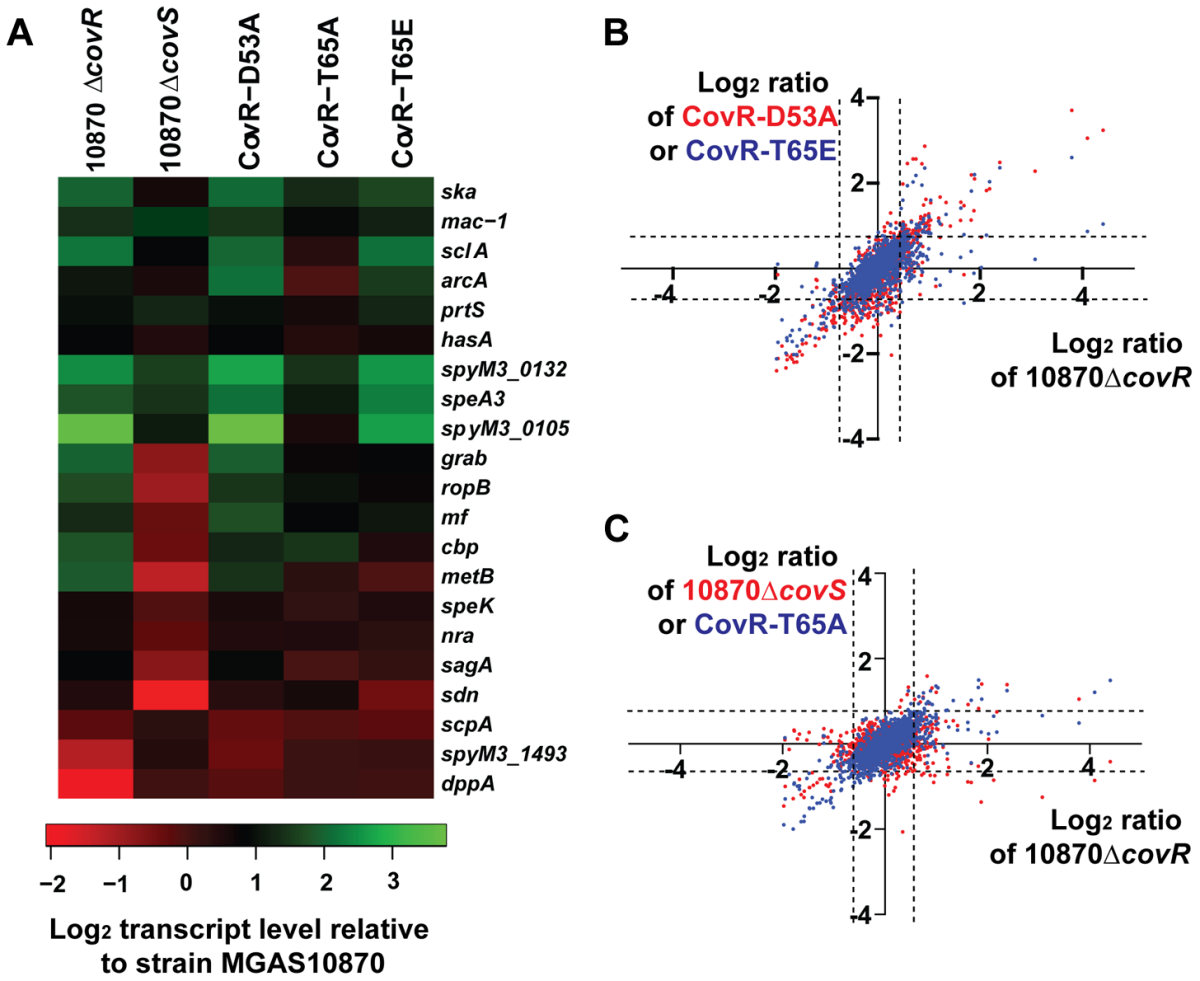
RNA-Seq analysis of various GAS strains. Six strains were grown in quadruplicate to mid-exponential phase. (A) Heat-map of log_2_ transcript levels of selected genes for the indicated strains relative to strain MGAS10870. Color value scheme is shown below the figure. (B) Pairwise comparison of log_2_ transcript values relative to the wild-type strain MGAS10870 for the indicated strains. Each dot represents a particular gene. Values for strain 10870Δ*covR* are plotted along the X-axis whereas values for strain CovR-D53A (red) and CovR-T65E (blue) are plotted along the Y-axis. The dotted lines indicate the 1.5-fold difference which was on e of our criteria for statistically significant transcript levels. (C) Transcript level data relative to strain MGAS10870 are plotted as in panel A with values for strain 10870Δ*covR* plotted along with X-axis and values for strain 10870Δ*covS* (red dots) and CovR-T65A (blue dots) plotted along the Y-axis.

Gene transcript levels were considered significantly different between the wild-type and a derivative strain if the mean transcript level had at least a 1.5-fold change and the multiple-testing adjusted *P* value was ≤0.05. Compared to wild-type, there were significant differences in transcript levels for 200 genes in strain 10870Δ*covR*, or some 10% of the genome (Table S1). Known CovR-influenced genes whose transcript levels were higher in strain 10870Δ*covR* included *hasA*, the initial gene in the hyaluronic acid capsule-encoding operon [45], *prtS*, which encodes an IL-8 degrading enzyme [46], and *grab*, which encodes the protein G-related α2-macroglobulin-binding protein [47] (Figure 5A). Genes with decreased transcript level in the CovR knockout strain included the dipeptide permease encoding operon (*dppA*-*E*) [48] and gene *spyM3_1493* which encodes a platelet acetyltransferase degrading enzyme known as esterase or Sse [49].

Consistent with D53 phosphorylation being critical to CovR function, the transcriptome of strain CovR-D53A was quite similar to that of the CovR-inactivated strain. Pairwise comparison of log_2_ transcript level ratios of strains CovR-D53A and 10870Δ*covR* relative to the wild-type strain MGAS10870 showed a Pearson correlation r^2^ value of 0.65 (*P*<0.001). The transcript levels of genes encoding virulence factors well-established to be repressed by CovR such as *hasA*, *prtS*, and *ska*, which encodes the plasminogen-activating streptokinase [50], were increased relative to wild-type in strain CovR-D53A (Figure 5A). However, the transcript levels of the dipeptide permease operon and the esterase-encoding gene *spyM3_1493*, although decreased in the CovR knockout strain, were not significantly altered compared to wild-type in the CovR-D53A strain (Figure 5A).

We previously found that changing T65 to glutamate (T65E) prevented phosphorylation of CovR at D53 by the small molecule phosphodonor acetyl phosphate *in vitro*. Thus we hypothesized that the CovR-T65E strain would have a transcriptome similar to the CovR-D53A strain. Indeed, among all the strains studied in this experiment, the Pearson correlation of log_2_ transcript levels ratios relative to the wild-type strain were the highest for strain CovR-D53A and CovR-T65E (r^2^ = 0.86) (Figure 5B). Genes whose transcript levels were significantly increased in strain CovR-T65E relative to wild-type included the key virulence factor-encoding genes *hasA*, *mac*-*1* (also known as *ideS*), which encodes an immunoglobulin degrading enzyme [51], *prtS*, and *sagA*, which encodes the cytolysin streptolysin S [52] (Figure 5A). Similar to strain CovR-D53A, we observed no difference in the transcript levels of the CovR-activated genes *dppA* and *spyM3_1493* between strain CovR-T65E and strain MGAS10870.

The 10870Δ*covR*, CovR-D53A and CovR-T65E strains each had approximately 200 genes with altered gene transcript levels relative to strain MGAS10870. In contrast, the replacement of CovR T65 with an alanine resulted in a strain with only 111 genes whose transcript levels were significantly different compared to wild-type (Figure 5C). The transcript levels of approximately half of the virulence factor encoding genes affected in the CovR-knockout strain were also affected in the CovR-T65A strain including *ska*, *mac-1*, and *prtS*. Genes whose transcript levels were affected in the CovR-knockout and CovR-D53A strains but not in strain CovR-T65A included *sagA* and *mf*, which encodes a DNA degrading enzyme [53]. As we had observed for strains CovR-D53A and CovR-T65E, there was no significant transcript level difference compared to wild-type for the CovR-activated genes *dppA* and *spyM3_1493* in strain CovR-T65A.

In contrast to the profound transcriptome effects of altering the CovR phosphorylation sites D53and T65, inactivating CovS had a relatively modest impact on GAS gene transcription during growth in THY (Figure 5C). Only 39 genes had significantly different transcript levels between the CovS-knockout and wild-type strain, which was the lowest number of differentially transcribed genes observed for any of the strains studied. Also, the effect of CovS inactivation led a distinct pattern of differential gene expression compared to the strains in which CovR had been altered. Specifically, in the CovR-isoallelic strains, all significant transcript level differences in CovR-repressed genes involved an increase in transcript levels relative to wild-type (Figure 5A). Conversely, CovS inactivation could result in either an increase or decrease in transcript level for CovR-repressed genes compared to wild-type, an effect that has been previously described for a CovS-inactivated serotype M1 strain [25]. For example, compared to strain MGAS10870, *mac-1*, *prtS*, and *ska* transcript levels were all significantly increased in strain 10870Δ*covS* whereas *ropB*, which encodes an transcription factor activating *speB* expression [54], and *grab* transcript levels were decreased (Figure 5A).

### CovR-T65A, but not CovR-T65E or CovR-D53A, retains the ability to repress gene expression in response to Mg^2+^

**Figure 6:**
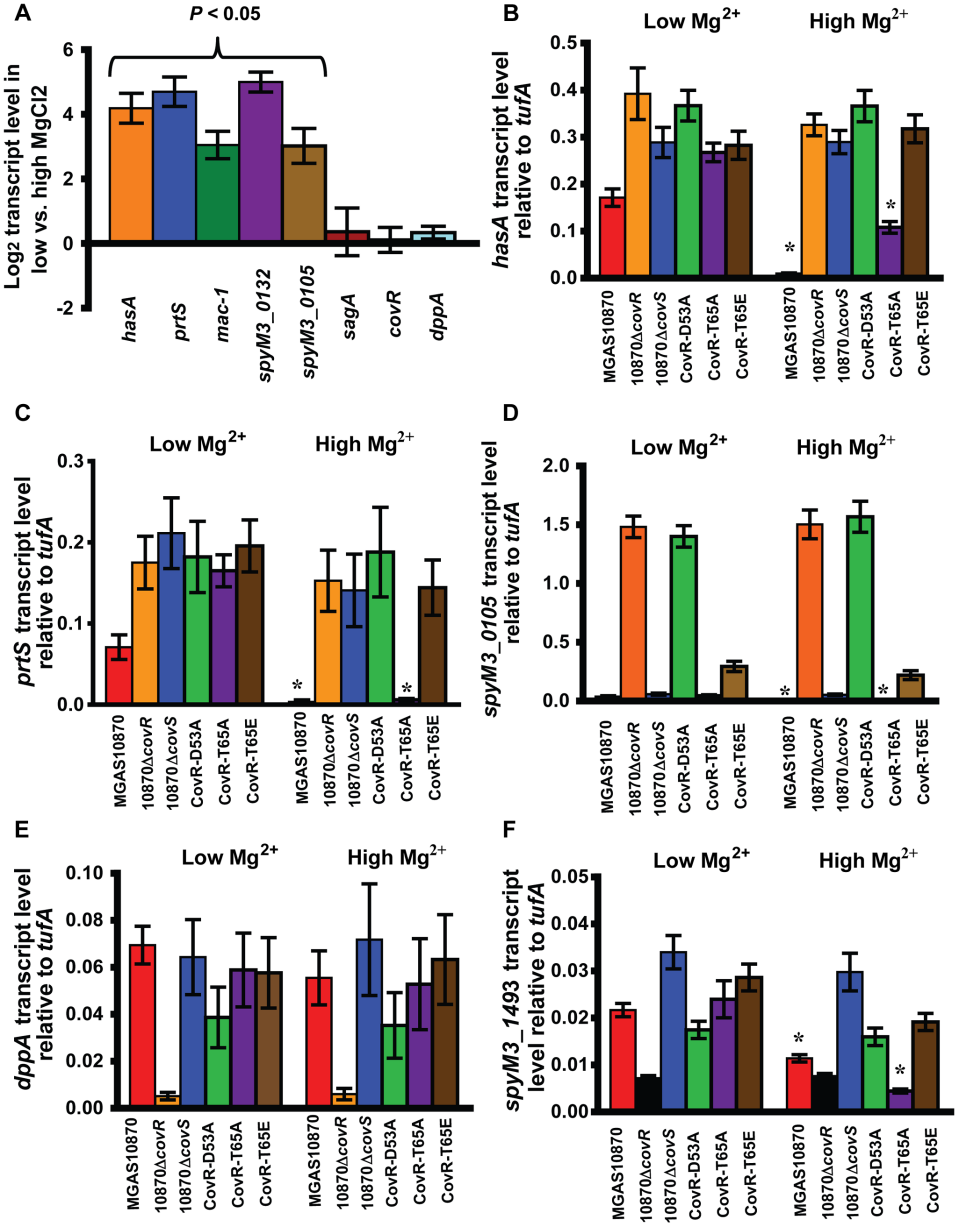
Strains MGAS10870 and CovR-T65A respond to changes in Mg^2+^. For all panels, data shown are gene transcript levels as measured by TaqMan quantitative real-time PCR (qRT-PCR). Strains were grown in normal THY (low Mg^2+^) or in THY with 15 mM MgCl_2_ (high Mg^2+^) to mid-exponential phase in duplicate on two separate occasions and analyzed in duplicate. Data shown are mean ± standard deviation of 8 data points. (A) Log_2_ ratio of transcript levels of indicated genes during growth in low vs. high Mg^2+^ medium in strain MGAS10870. *P* value refers to Student's t-test comparing gene transcript levels in low vs. high Mg^2+^ medium. (B–F) Data shown are transcript levels of indicated genes in indicated strains relative to the endogenous control gene *tufA*. * indicates significant difference in gene transcript for the indicated strain between growth in a low Mg^2+^ vs. high Mg^2+^ conditions as determined by Student's t-test.

Adding Mg^2+^ to standard laboratory medium is known to increase the repression of a subset of CovR-regulated genes, presumably by increasing CovS-mediated CovR D53 phosphorylation (Figure 1C) [41]. Thus, we next sought to specifically analyze the response of CovR-regulated genes to Mg^2+^ in our isogenic and isoallelic strains using quantitative real-time PCR (qRT-PCR). In accordance with previous data, in the wild-type strain MGAS10870, we observed significantly higher gene transcript levels during growth in a low Mg^2+^ medium compared to a high Mg^2+^ medium for *hasA*, *prtS*, *mac*-*1*, *spyM3_0132*, and *spyM3_0105* (Figure 6A) [41], [55]. This effect did not appear to be due to altered CovR levels as there was no significant difference in *covR* gene transcript levels between the two media (Figure 6A). Similarly, we observed no significant differences in transcript levels between the two media for *sagA* or *dppA* (Figure 6A). Consistent with the idea that CovS phosphorylation on CovR D53 mediates the response to Mg^2+^, we saw no difference in *hasA*, *prtS*, or *spyM3_0105* gene transcript levels in either strain 10870Δ*covR*, 10870Δ*covS*, CovR-D53A, or CovR-T65E between growth in low and high Mg^2+^ concentration (Figure 6B–D). Conversely, for strains MGAS10870 and CovR-T65A, the gene transcript levels of *hasA*, *prtS*, and *spy_0105* were significantly decreased in the high Mg^2+^ medium consistent with the hypothesis that the CovR-T65A strain can still be phosphorylated on D53 *in vivo*. We next examined the CovR-activated genes *dppA* and *spyM3_1493*. There was no difference in *dppA* transcript level in response to altered Mg^2+^ concentration for any of the studied strains (Figure 6E) whereas there was a significant decrease in the transcript level of *spyM3_1493* in strains MGAS10870 and CovR-T65A (Figure 6F) under high Mg^2+^ condition. Thus, of the strains tested here, a Mg^2+^ response for CovR-regulated genes was only observed for strains MGAS10870 and CovR-T65A.

### Responsiveness to Mg^2+^ is independent of DNA binding affinity of CovR-D53∼P

The finding that some, but not all CovR-regulated genes, have altered gene transcript levels in the presence of increased Mg^2+^ concentration has previously been noted [41], but the explanation for this observation is not known. One possibility that has been postulated is that Mg^2+^-responsive promoters are bound by CovR phosphorylated at D53 (CovR-D53∼P) with lower affinity compared to Mg^2+^ non-responsive promoters [41]. Consequently, Mg^2+^-responsive genes would only be fully repressed by CovR under conditions that increase CovR phosphorylation above that observed in a standard laboratory medium (compare Figure 1C and 1D for different CovR phosphorylation levels under different growth conditions). To test this hypothesis, we measured the DNA binding properties of wild-type CovR-D53∼P for the Mg^2+^ non-responsive promoters *covR* and *dppA* in comparison to the Mg^2+^ responsive promoters *prtS* and *hasA*. Unexpectedly, we observed that CovR-D53∼P bound to DNA from both groups with similar affinity (Figure S4 in Text S1). For all promoters tested, CovR-D53∼P mediated shifts began at CovR concentration of 0.1–0.2 µM to form a low molecular weight complex; at ∼1 µM the higher molecular weight complex became more prominent. Hence, *in vitro* DNA affinity alone does not seem to be the decisive factor for the different response of CovR regulated promoters towards Mg^2+^ ions. However, these data do not rule out differential CovR-DNA affinity *in vivo* given the complex nature of CovR-DNA interaction in the bacterial cell [16].

### Recombinant CovR-D53A and CovR-T65E retain the ability to bind promoter DNA from CovR-regulated genes

**Figure 7:**
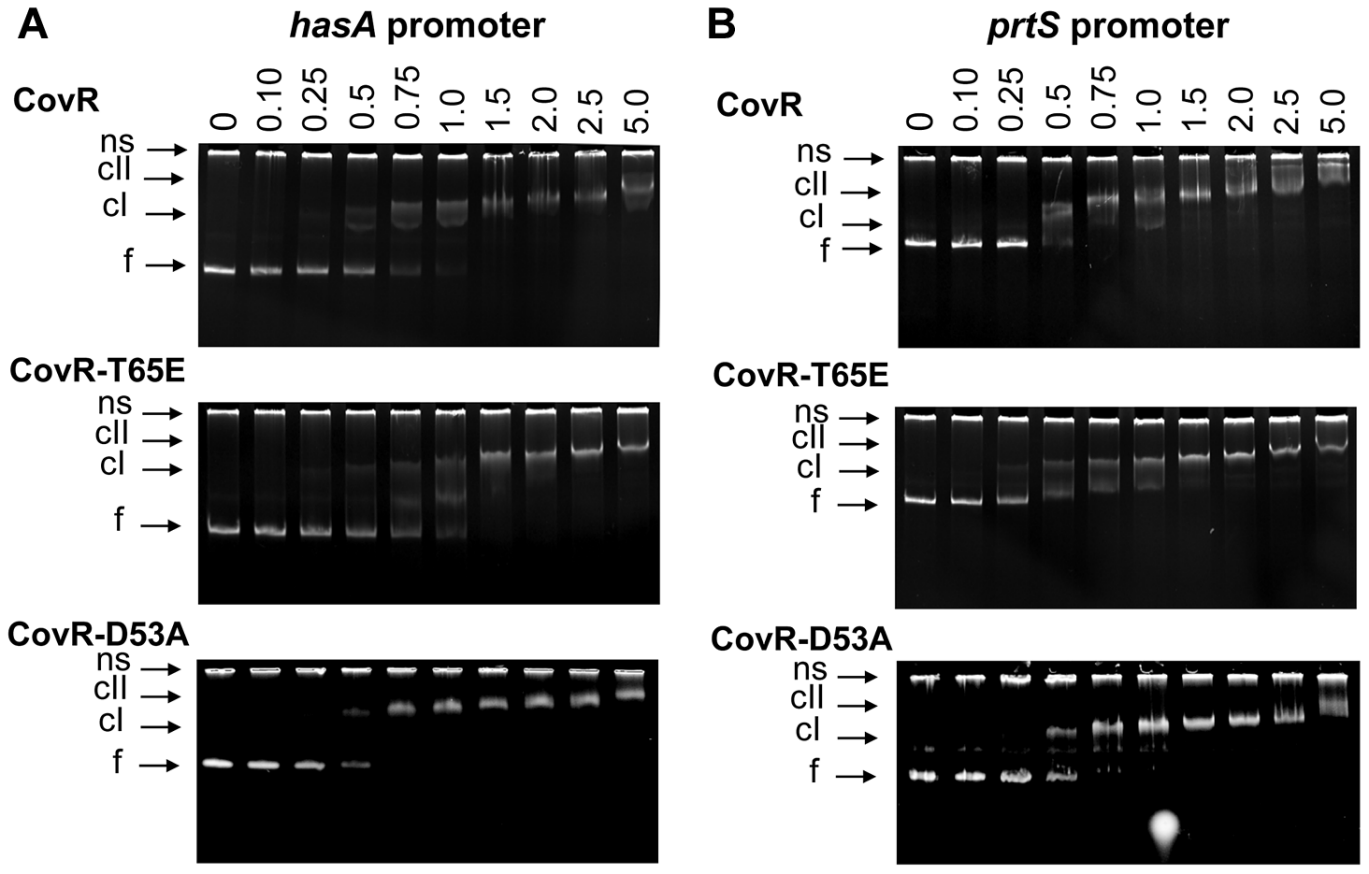
CovR-D53A and CovR-T65E bind CovR-regulated promoters with similar affinity to unphosphorylated CovR. Binding of recombinant CovR, CovR-D53A, and CovR-T65E proteins to the promoters of (*A*) *hasA* and (B) *prtS*. Increasing concentrations of unphosphorylated CovR, CovR-D53A, and CovR-T65E (monomer concentration given in µM) were used as indicated. Samples were incubated for 15 min at 37°C and electrophoresed on a 6% TBE polyacrylamide gel for 60 min at 120 V. The gels were stained with ethidium bromide. ns, non-specific DNA, f, free DNA, cI, lower molecular weight complex, cII, higher molecular weight complex. Gels shown are representatives of identical results obtained on two separate occasions.

We hypothesize that the primary functional effect of CovR T65 phosphorylation is to prevent phosphorylation at D53, and a previous study found that recombinant CovR-D53A bound to DNA from the *has* promoter with the same affinity as unphosphorylated wild-type CovR [22]. However, in group B *Streptococcus*, recombinant CovR-T65E was unable to bind to DNA from the promoter of CovR-regulated gene *cylX* [29]. Thus, we sought to determine the DNA binding characteristics of GAS CovR-T65E compared to wild type CovR and CovR-D53A. To this end, ∼300 bp DNA fragments that encompass the complete promoter regions of the CovR-regulated genes *hasA, prtS*, and *sagA* were amplified from MGAS10780 genomic DNA [33], [46], [56]. Both CovR-D53A and CovR-T65E bound to all of the tested promoters (Figure 7 and Figure S5 in Text S1). The binding affinity of CovR-D53A and CovR-T65E was similar to that of unphosphorylated wild-type CovR, with initial shifts starting at 0.25–0.5 µM protein concentration (formation of a low molecular weight complex) and a full shift at ∼1.5 µM protein concentration (Figure 7). The specificity of CovR DNA binding was ascertained using a promoter that is not CovR-regulated, *adcR* (Figure S5 in Text S1).

### Phosphorylation of CovR significantly affects GAS virulence and gene expression during infection

**Figure 8:**
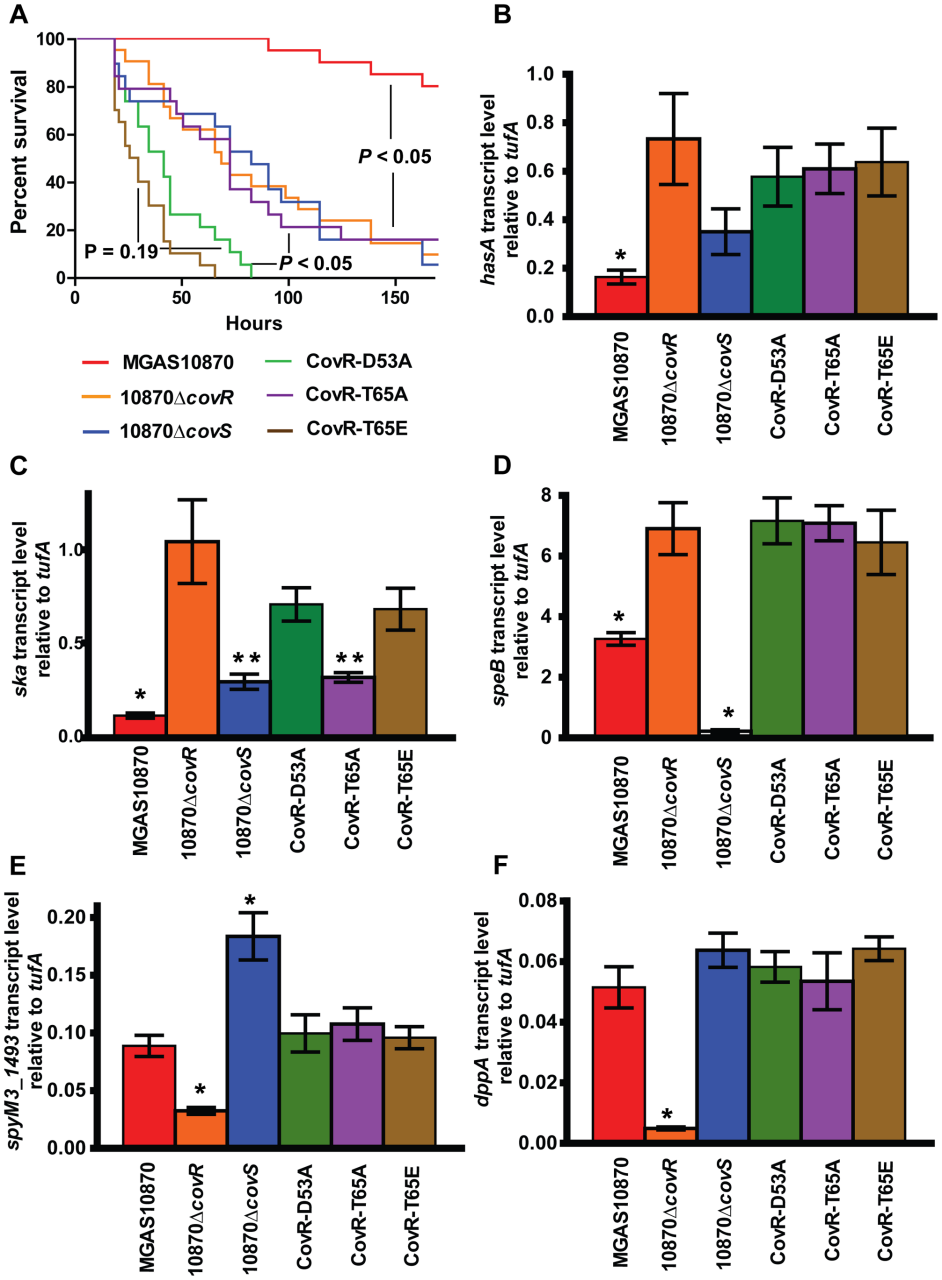
CovR phosphorylation affects GAS virulence and gene expression during infection. (A) 20 CD-1 mice per strains were challenged intra-peritoneally with 1×10^7^ colony forming units of the indicated strains and followed for near-mortality. Data graphed are survival. *P* values refer to Mantel-Cox (log-rank) test adjusted for multiple comparisons. For (B–F), RNA was isolated from infected animals as described in the Material and Methods and converted to cDNA. Data graphed are gene transcript levels of the indicated genes relative to the endogenous control gene *tufA*. Four animals were infected with each strain and data were analyzed in triplicate. Data graphed are mean ± standard deviation of 12 data points. * indicates that gene transcript level for that particular strain was significantly different compared to all other strains as determined by analysis of variance (ANOVA) and Bonferroni's post-hoc test. For panel (C), ** indicates that the transcript level of *ska* was significantly lower in strains 10870Δ*covS* and CovR-T65A compared to strains 10870Δ*covR*, CovR-D53A, and CovR-T65E.

To test whether CovR phosphorylation affects GAS virulence, outbred CD-1 mice were challenged intraperitoneally with the various CovS- or CovR-inactivated and CovR isoallelic strains and followed for near-mortality over 7 days. As expected, there was a significant survival difference amongst the mice challenged with the 6 strains (*P*<0.001 by Mantel-Cox log rank test, Figure 8A). Specifically, strain MGAS10870 was less virulent compared to the other five strains (*P*<0.001 after accounting for multiple comparisons). There was no significant difference in survival among mice infected with strains 10870Δ*covR*, 10870Δ*covS*, or CovR-T65A (*P* = 1.0). However, the survival time was significantly shorter for mice infected with strains CovR-D53A or CovR-T65E compared to strains 10870Δ*covR*, 10870Δ*covS*, or CovR-T65A (*P*<0.01 for all comparisons). No significant difference in survival was observed between mice infected with strain CovR-D53A or CovR-T65E (*P* = 0.19).

We sought to gain insight into the survival differences noted in the animal challenge experiment by determining the gene expression profile of bacteria during infection. To this end, we euthanized 4 animals per infecting strain 24 hours after infection, isolated GAS RNA from the blood of infected animals, and analyzed select gene transcript levels. As expected, the transcript levels of *hasA*, *sagA*, and *prtS* genes were significantly higher in the CovR-inactivated and CovR-isoallelic strains compared to wild-type (Figure 8B, data not shown). The transcript levels of *ska* were significantly lower in the wild-type strain compared to the other strains, but *ska* transcript levels were also lower in strain 10870Δ*covS* and CovR-T65A compared to strains 10870Δ*covR*, CovR-D53A, and CovR-T65E (Figure 8C). Similarly to what we had observed during growth in THY, inactivation of CovS significantly decreased *speB* transcript level and significantly increased *spyM3_1493* transcript level compared to wild-type and the other tested strains (Figure 8D, 8E). Moreover, we observed decreased transcript level of *spyM3_1493* and *dppA* specifically in strain 10870Δ*covR* compared to the other tested strains (Figure 8E, 8F).

### CovR amounts *in vivo* are lower in the CovR-T65A isoalleic strain compared to wild type

**Figure 9:**
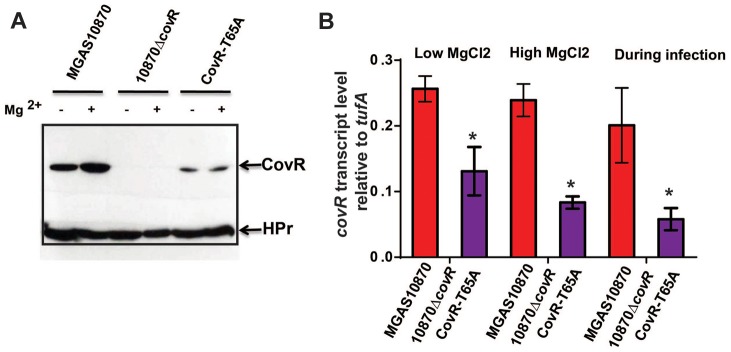
CovR levels are decreased in the CovR-T65A strain. (A) Western blot analysis. Indicated strains were grown to mid-exponential phase in regular THY medium (−) and in THY medium supplemented with 15 mM MgCl_2_ (+). Cell lysates containing 70 µg of total protein were loaded on a 12% SDS-gel. The gel was divided into two parts; the upper part was blotted against anti-CovR antibody while the lower part was blotted against anti-HPr antibody which served as a loading control. (B) qRT-PCR analysis of *covR* transcript levels. For “Low MgCl_2_” and “High MgCl_2_” samples, the indicated strains were grown as for the Western blot analysis in duplicate on two separate occasions and analyzed in duplicate. Data shown are mean ± standard deviation of 8 data points. For the “During infection” samples, four animals were infected with each strain and data were analyzed in triplicate as described in Figure 8. Data graphed are mean ± standard deviation of 12 data points. For all three conditions the lack of a bar for 10870Δ*covR* strain indicates no *covR* transcript level was detectable. * designates a significant difference in *covR* transcript level between strain MGAS10870 and CovR-T65A as determined by Student's t-test.

Our *in vitro* experiments showed that mutation of CovR T65 to alanine impedes Stk-mediated phosphorylation, while having no impact on phosphorylation of D53 by acetyl-phosphate. Hence, we predicted that the concentration of CovR-D53∼P in the CovR-T65A strain would be equal or even higher than in the parental wild type strain leading to pronounced CovR-mediated gene repression and decreased virulence. However, our expression and animal data did not confirm this hypothesis as the CovR-T65A strain showed increased virulence compared to strain MGAS10870. As CovR is known to repress its own expression [57], we compared the CovR transcript and protein levels in wild type strain MGAS10780, the CovR-deletion strain, and the CovR-T65A isoallelic strain using qRT-PCR and Western blot analysis, respectively. As expected no CovR signal was detected in strain10870Δ*covR* (Figure 9A). However, the amount of CovR was significantly higher in the wild type compared to the CovR-T65A strain under both high and low Mg^2+^ growth conditions (Figure 9A). Similarly, the level of *covR* transcript was significantly higher in strain MGAS10870 compared to the CovR-T65A isoallelic strain (Figure 9B). Finally, during infection, we also observed significantly decreased *covR* transcript levels in the CovR-T65A strain compared to strain MGAS10870 (Figure 9B). Consequently, at least part of the characteristics of the CovR-T65A strain is likely a result of decreased *covR* expression due to increased CovR self-repression and thus lower CovR levels compared to wild-type.

## Discussion

Until recently, studies of how protein phosphorylation participates in prokaryotic signal transduction have mainly focused on linear TCS pathways that involve phosphorylation of aspartate in response regulators by their cognate histidine kinase [9]. Increasingly, however, it is being recognized that the phosphorylation status of response regulators can be influenced by a multitude of factors, including phosphorylation on non-aspartate residues, clearly placing response regulators into the wider gene regulation network [28], [58], [59]. Understanding the mechanisms influencing the phosphorylation status of bacterial regulator proteins and determining how such phosphorylation ultimately influences bacterial gene expression is critical to a more complete understanding of bacterial pathogenesis. Herein we demonstrate that the key GAS response regulator CovR is phosphorylated *in vivo* and that CovR phosphorylation at both aspartate and threonine residues profoundly influences GAS global gene expression and virulence.

The recognition of CovR in 1998 as a negative regulator of GAS hyaluronic acid capsule production was accompanied by the observation that CovR is a OmpR/PhoB family member, and as such, contains a highly conserved aspartate phosphorylation site [15]. It was also determined at that time that the *covR* gene is located immediately upstream of *covS* and that the two genes are co-transcribed, although mono-cistronic *covR* transcripts have also been reported [15], [60]. This combination of observations has led to the supposition that CovS controls CovR aspartate phosphorylation status analogous to the situation observed for the well-studied EnvZ/OmpR TCS in *Escherichia coli*, although CovS has never been directly shown to phosphorylate CovR *in vitro* or *in vivo* [16]. Over the past 15 years, numerous investigations have demonstrated the critical role of CovR D53 phosphorylation in CovR-mediated gene repression, mainly relying on *in vitro* binding assays and transcriptional reporter systems [22], [32], [56], [57]. However, up to now, demonstration of CovR phosphorylation *in vivo* has been lacking. Our finding that a Phos-Tag assay can be used to measure GAS and GBS CovR phosphorylation status *in vivo* opens extensive possibilities into understanding the mechanisms and impact of streptococcal response regulator phosphorylation. Moreover, the fact that the CovS-inactivated and CovR-D53A strains had dramatically different transcriptomes provides additional impetus to study which factors, other than CovS, influence CovR D53 phosphorylation.

In addition to demonstrating CovR phosphorylation *in vivo*, another key finding of this work was the identification of T65 as the target of Stk phosphorylation in CovR using both mass spectrometry and targeted mutagenesis strategies. A previous work had established that recombinant Stk phosphorylates CovR on threonine residues but did not establish the phosphorylation site [26]. In contrast to the highly specific phosphotransfer in TCS, eukaryotic-like serine/threonine kinases, such as Stk, are well known to have multiple targets, including cell-wall division regulating proteins that are critical to cellular homeostasis [61], [62], [63]. Thus, genetic inactivation of Stk or its cognate phosphatase, Stp, as has previously been done in serotype M1 GAS strains, is unlikely to generate information specifically relevant to threonine phosphorylation of CovR [42], [64]. For example, in serotype M1 GAS strains, inactivation of both Stk and its cognate phosphatase, Stp, resulted in decreased virulence, probably as a result of their pleiotropic effects on GAS physiology [26], [64]. Thus, once we established the site of CovR-threonine phosphorylation, we used a combination of deletion (Δ*covR*, Δ*covS*), phosphorylation silencing (CovR-D53A and CovR-T65A), and phosphorylation mimicking (CovR-T65E) mutations to comprehensively assess the functional consequences of CovR phosphorylation. Our data indicate that the combination of competing CovR D53 and T65 phosphorylation enables the integration of thus far unknown signals sensed by Stk into the regulatory network in order to fine-tune CovR/S-mediated gene regulation during GAS host-pathogen interaction.

One surprising finding from this study was that the CovR-D53A and CovR-T65E strains were more virulent compared to strain 10870Δ*covR*. A likely explanation for this finding can be gleaned from our transcriptome data. Whereas CovR-mediated repression of gene expression strongly depended on phosphorylation of D53 (which is abolished in strains CovR-D53A and CovR-T65E), it had minimal effect on CovR-mediated gene activation. A possible target to explain the virulence effects of this differential gene regulation is *spyM3_1493*, which encodes a platelet-activating factor acetylhydrolase recently shown to be critical for GAS pathogenesis [65]. Review of previously published microarray data from serotype M3 GAS confirms that *spyM3_1493* transcript levels were lower in CovR-inactivated strains compared to the wild-type [41], [43]. Similar findings of lower transcript levels in the CovR-inactivated, but not the CovR-D53A and CovR-T65E, strains were also observed for the *dpp* operon, which encodes proteins involved in amino acid uptake [48]. Apparently, merely the presence of CovR, not its phosphorylation status, is crucial for the regulation of these genes. The mechanism of CovR-mediate gene activation is unknown as neither CovR nor CovR-D53∼P was sufficient to activate transcription from the *dppA* promoter *in vitro* despite the fact that CovR specifically binds to the *dppA* promoter [48]. Thus, whereas nearly all previous GAS CovR related work has focused on CovR-mediated gene repression, our data provide new impetus to better elucidate the mechanism underlying CovR gene activation. Moreover, these data may provide an explanation for why the majority of naturally occurring CovR variation identified in strains causing invasive infection involves alterations in N-terminal amino acid residues, rather than truncations of the CovR protein as are commonly observed for CovS [43], [66].

T65 has also previously been identified as the site for Stk-mediated phosphorylation of CovR in GBS [29]. However, despite significant parallels, such as the site of phosphorylation and negative interplay between the two phosphorylation sites [28], [29], [30], there are some striking differences between GAS and GBS in terms of the effect of CovR T65 phosphorylation. For example, GBS CovR-T65E was completely unable to bind to DNA from the CovR-regulated *cylX* promoter [29]. In contrast, we found that recombinant GAS CovR-T65E and CovR-D53A bound DNA from several CovR-regulated promoters at concentrations equivalent to unphosphorylated wild type CovR, which is consistent with our transcript data showing that the CovR-T65E and CovR-D53 strains maintained CovR-mediated gene activation profiles. Thus, in GAS, it appears that Stk-mediated phosphorylation of CovR T65 reduces, but does not completely abrogate CovR-DNA binding as was observed in GBS. Given that GAS CovR D53 phosphorylation increases interaction with RNA polymerase in addition to enhancing CovR-DNA interaction [22], GAS CovR T65 phosphorylation likely has additional consequences besides affecting CovR DNA binding affinity. Another key difference between the CovR system in GAS and GBS is that GAS CovR represses its own transcription whereas GBS CovR is self-activating [30], [57]. There is significant variance in the *covR* promoter regions between GAS and GBS, including differences at previously identified key GAS CovR binding sites [57]. Abolishing CovR threonine phosphorylation in GBS via the T65A substitution increased *covR* transcript level whereas we observed decreased *covR* transcript and decreased CovR expression in GAS CovR-T65A [30]. Interestingly, in GBS the CovR-T65A variant was also hypervirulent in mice compared to wild-type and hypovirulent compared to the CovR-D53A and CovR-T65E strains, which was the same phenotypic hierarchy we observed in GAS [30].

These and other data on CovR-Stk interaction in GAS and GBS raise the important question of how T65 phosphorylation hampers D53 phosphorylation and *vice versa* [26], [42]. The best studied example of dual-site phosphorylation in a prokaryotic pathogen concerns the histidine-containing phosphocarrier (HPr) protein, which can be phosphorylated on histidine-15 as well as serine-46 [67]. The various phosphorylation states of HPr regulate its interaction with other proteins as exemplified by the fact that HPr-S46-P, but not HPr-H15∼P can interact with the regulatory protein catabolite control protein A (CcpA) [68]. Conversely, HPr-His15∼P, but not HPr-S46-P serves as component of the phosphorelay in the phosphoenol-phosphotransferase system (PTS) regulating PTS-sugar transport [67]. In contrast to HPr, it does not appear that the phosphorylated forms of CovR are active in distinct functional systems but instead serve to influence the final effect of CovR on gene expression. Rather than obstructing specific protein-protein interactions (e.g. CovR-CovS), it is likely that CovR T65 phosphorylation induces an allosteric change in the CovR N-terminal receiver domain that serves to inhibit D53 phosphorylation by interfering with the general activation mechanism for OmpR/PhoB family members [69]. Such a change would explain why even the small molecule phosphodonor acetyl phosphate is unable to phosphorylate CovR-T65E. Similarly, although the CovR homolog OmpR is not known to be regulated by serine/threonine phosphorylation, fluorescence-labeling of OmpR cysteine-67, which corresponds to T65 in CovR, also impaired OmpR aspartate-phosphorylation and *vice versa* [70]. A crystal structure of the N-terminal domain of CovR is not available, but modeling of CovR on the structure of the related response regulator PhoP in complex with the phosphomimic BeF_3_^−^ (PBD code 2PL1) shows that D53 and T65 are ∼9 Å apart meaning that interference due to direct electrostatic repulsion can be excluded [71]. We are currently engaged in generating crystal structures of the N-terminal domain of CovR and its various phosphorylated forms in an attempt to better understand the molecular consequences of CovR dual-site phosphorylation.

In summary, we have employed a combination of biochemical, genetic, and virulence assays to generate new insights into how phosphorylation of CovR mediated by distinct pathways affects GAS pathogenesis. Further studies of how bacteria modulate the phosphorylation status of regulatory proteins may generate new opportunities to prevent or treat serious bacterial infections.

## Materials and Methods

### Ethics statement

This study was carried out in accordance with the recommendations in the Guide for the Care and Use of Laboratory Animals of the National Institutes of Health. The protocol was approved by MD Anderson Animal Care and Use Committee (Protocol Number: 07-09-09131). All efforts were made to minimize suffering.

### Bacterial strains and culture media

The strains and plasmids used in this work are presented in Table 1, and primers used for strain creation are listed in Table S2. Strains were grown in a nutrient-rich standard laboratory medium (Todd-Hewitt broth with 0.2% yeast extract (THY)) at 37°C with 5% CO_2_ with 15 mM MgCl_2_ added when noted to create a high Mg^2+^ condition [41]. THY without supplemental MgCl_2_ has previously been determined to have a Mg^2+^ concentration of ∼1 mM [72]. When appropriate, spectinomycin and chloramphenicol were added at 150 µg/mL and 10 µg/mL, respectively. Strain MGAS10870 is a fully-sequenced, invasive, serotype M3 strain that contains a wild-type *covRS* operon [40]. Strains 10870Δ*covR* and 10870Δ*speB* have been previously described [43], [73]. Strain 10870Δ*covS* was created by replacing the *covS* gene in strain MGAS10870 with a spectinomycin resistance cassette via insertional inactivation as described [17], [25]. Derivatives of strain MGAS10870 that differed only by the presence of a single amino acid replacement were created using the chloramphenicol-resistant, temperature-sensitive plasmid pJL1055 (gift of D. Kasper) as described [74]. For each of the CovR strains derived from strain MGAS10870, sequencing of the entire *covRS* operon, as well as the *mga* and *emm* genes, was performed to check for the presence of spurious mutations that could have developed during the strain construction process (none were found). Strain SGBS001 is a serotype V group B *Streptococcus* strain isolated from human blood in Houston.

**Table 1:** Strains and plasmids used in this work.

Strain or plasmid	Description	Reference
**Strains**		
MGAS10870	Invasive isolate, serotype M3, CovR wild-type	[40]
10870Δ*covR*	MGAS10870 Δ*covR*::*aphA3*	[39]
CovR-D53A	MGAS10870 with *covR* encoding Ala at 53	This study
CovR-T65A	MGAS10870 with *covR* encoding Ala at 65	This study
CovR-T65E	MGAS10870 with *covR* encoding Glu at 65	This study
10870Δ*covS*	MGAS10870 Δ*covS*::*spc*	This study
10870Δ*speB*	MGAS10870 ΔspeB::spc	[73]
SGBS001	Serotype V group B *Streptococcus*	This study
**Plasmids**		
pJL1055	Low-copy number shuttle vector with temperature-sensitive replication in GAS	[80]
pSSCovR-D53A	pJL1055 with *covR* encoding Ala at 53	This study
pSSCovR-T65A	pJL1055 with *covR* encoding Ala at 65	This study
pSSCovR-T65E	pJL1055 with *covR* encoding Glu at 65	This study
pTXB1-CovR	pTXB1 with wild-type *covR*	[39]
pTXB1-CovR-D53A	pTXB1 with *covR* encoding Ala at 53	This study
pTXB1-CovR-T65A	pTXB1 with *covR* encoding Ala at 65	This study
pTXB1-CovR-T65E	pTXB1 with *covR* encoding Glu at 65	This study
pTXB1-CovR-T73A	pTXB1 with *covR* encoding Thr at 73	This study
pET15b-GAS-Stk_KD_	pET15b expressing the Stk kinase domain	This study

### Protein overexpression and purification

Recombinant CovR was isolated from plasmid pTXB1-*covR* using the IMPACT Protein Purification System (New England Biolabs), which allows for recovery of soluble CovR isoforms lacking any non-native residues (e.g. no His-tag) [39]. To generate CovR variants, single nucleotide exchanges were introduced into pTXB1-*covR* via Quick-change mutagenesis (Stratagene) using the respective primer pairs (see Table S2). Wild type and variant CovR proteins were overexpressed in *E. coli* BL21/pLysS at 18°C overnight and purified to ≥95% homogeneity as described previously [39]. All CovR proteins were extensively buffer exchanged to 50 mM CAPS pH 10.0, 100 mM NaCl. Purified CovR protein was used to immunize rabbits to generate anti-CovR antibody (Covance, Denver, PA). The specificity of the anti-CovR antibody for CovR is shown in Figure S2.

The isolated Stk kinase domain (Stk_KD_) has been shown previously to be sufficient to phosphorylate CovR [26]. Thus, recombinant Stk_KD_ was generated by amplifying DNA encoding the Stk kinase domain (amino acids 1–315) from MGAS10870. The resulting PCR product was cloned into pET15b (Novagen) via *Nde*I and *Xho*I. The N-terminal His-tagged Stk_KD_ was overexpressed in *E. coli* BL21/pLysS. Cells were induced with 1 mM IPTG at an OD ∼0.6, incubated for five hours at 20°C and harvested by centrifugation. After lysing the cells in buffer containing 20 mM Tris/HCl pH 8.0, 200 mM NaCl, 20 mM imidazole, and 10% glycerol, Stk_KD_ protein was purified over a Ni-NTA column and eluted with 200 mM imidazole. The protein was concentrated to ∼10 mg/ml and stored at −20°C in 20 mM Tris/HCl pH 8.0, 150 mM NaCl, 40% glycerol. The resultant Stk_KD_ protein was ∼% pure (Figure S1A).

### *In vitro* phosphorylation of CovR and detection of the phosphorylation status

Wild type and variant CovR proteins were phosphorylated on D53 for 2 h at 37°C in phosphorylation buffer containing 50 mM Tris/HCl pH 8.0, 10 mM MgCl_2_, 3 mM DTT, and 32 µM acetyl-phosphate (Sigma), as previously described [56]. Threonine phosphorylation of CovR was performed by incubating wild type or variant CovR proteins for 30 min at 37°C with a three-fold excess of Stk_KD_ in phosphorylation buffer containing 100 mM Tris/HCl pH 7.5, 10 mM MgCl_2_ or MnCl_2_, 1 mM DTT, and 10 mM ATP. To detect phosphorylated CovR species we used Phos-tag SDS polyacrylamide (PAA) gels which contain a phosphate-binding reagent that specifically retards the migration of phosphorylated proteins, thereby allowing for resolution between phosphorylated and non-phosphorylated protein species [35]. The phosphorylation samples were separated on 12% Phos-tag SDS-PAA gels containing 100 µM Phos-tag solution (Wako Pure Chemical Industries Ltd, Richmond, VA) and 200 µM MnCl_2_ for 70 min at 180 V. The gel was washed in transfer buffer (SDS running buffer containing 20% MeOH and 1 mM EDTA) for 15 min. The proteins were then transferred onto a nitrocellulose membrane (0.45 µm, Bio-Rad) using a Trans-blot SD semi-dry electrophoretic transfer cell (Bio-Rad) for 15 min at 15 V. After blocking the membrane overnight in 20 mM Tris/HCl pH 7.5, 150 mM NaCl, 0.05% Tween 20, and 5% dry milk, it was incubated for 1 h with affinity purified anti-CovR antibody at a 1∶5,000 dilution with blocking solution. The secondary antibody, goat anti-rabbit IgG (Pierce), was diluted 1∶10,000. The blot was developed using the SuperSignal West Pico Chemiluminescent Substrate (Thermo Scientific).

### Detection of CovR phosphorylation *in vivo*

GAS and GBS strains were grown in 100 ml of THY or THY supplemented with 15 mM MgCl_2_ to OD = 0.5 and harvested by centrifugation. Cell lysates were prepared by the rapid preparation method previously described taking care to always keep the lysates chilled to minimize spontaneous dephosphorylation [36]. A total protein amount of 70 µg as determined by Bradford assay (Bio-Rad) was loaded on the gel for each sample. As phosphorylation controls, wild type cell lysates were either boiled at 100°C for 1 min, which specifically removes the heat labile aspartate phosphorylation, or incubated with 40 units calf intestine phosphatase (CIP, New England Biolabs) at 37°C for 10 min, which completely removes any phosphorylation. Samples were electrophoresed for 80 min at 150 V on a 10% Bis-Tris buffered Zn^2+^-Phostag SDS-PAGE. This allows the separation of unphosphorylated and phosphorylated proteins under neutral pH [38]. Subsequently, CovR species were detected by standard Western blotting using anti-CovR antibodies.

### Circular dichroism spectroscopy

Wild type and mutant CovR proteins were diluted to a concentration of 0.4 mg/ml in 10 mM potassium phosphate, pH 7.5, 10 mM NaF prior to measurements. Far-UV (200–260 nm) spectra were recorded on a Jasco J-810 spectropolarimeter at 37°C and a scan speed of 20 nm/min as described [43].

### Mass spectrometry

15 µg of recombinant CovR was phosphorylated using Stk_KD_ as described above. The entire reaction was run on a 12% SDS polyacrylamide gel. The band corresponding to CovR was excised and 50 pmol of *in vitro* phosphorylated CovR was diluted into 45 µl of 0.1 M ammonium bicarbonate, reduced with 2 µl 0.1 M DTT and alkylated with 2 µl 0.2 M iodoacetamide. The protein was digested with 1 µg of chymotrypsin (Roche, Indianapolis, IN) at 25°C or 1 µg of trypsin at 37°C overnight. 10 µl of the peptide mixture was analyzed by automated microcapillary liquid chromatography-tandem mass spectrometry (LC-MS-MS). The application of a 1.8 kV distal voltage electro-sprayed the eluted peptides directly into the LTQ Orbitrap XL ion trap mass spectrometer equipped with a nanoLC electrospray ionization source (ThermoFinningan). Full masses (MS) spectra were recorded on the peptides over a 400–2000 *m*/*z* range at 60,000 resolution in the Orbitrap, followed by five tandem mass (MS/MS) events sequentially generated in a data-dependent manner on the first, second, third, fourth and fifth most intense ions selected from the full MS spectrum (at 35% collision energy). Mass spectrometer scan functions and HPLC solvent gradients were controlled by the Xcalibur data system (ThermoFinnigan). MS/MS spectra were extracted from the RAW file with ReAdW.exe (http://sourceforge.net/projects/sashimi). The MS/MS data were searched with Inspect [75] against the MGAS315 *Streptococcus pyogenes* database (NC_004070) with optional modifications: +15.9994 on methionine, +57.0214 on cysteine, and +79.9663 on threonine, serine, and tyrosine, respectively. Only peptides with at least a *P*-value of 0.01 were analyzed further. Data from keratins and trypsin were excluded from further analysis. Peptides identified by the software to be phosphorylated were manually verified. In addition, further verification was performed by analyzing putative phosphorylated peptides in targeted MS/MS and MS/MS/MS modes.

### Hyaluronic acid capsule and cysteine protease activity assays

The amount of cell-associated capsular polysaccharide was done as previously described using GAS grown to late-exponential phase (OD_600_ = 0.9) [15]. Functional SpeB protease activity was determined using casein hydrolysis as described [76].

### Transcript level analysis

RNA was purified from various GAS strains grown to mid-exponential phase using an RNeasy mini kit (Qiagen). 1 µg of RNA per sample was converted to cDNA using a High Capacity Reverse Transcription Kit (Applied Biosystems). TaqMan real-time qRT-PCR (primers and probes listed in Table S2) was performed on an Applied Biosystems Step-One Plus System as described [77]. All samples were done at least in duplicate on two separate occasions and analyzed in duplicate. To compare gene transcript levels between the wild-type and the various derivative strains, a two-sample *t*-test (unequal variance) was applied with a *P* value of <0.05 and a mean transcript level of at least 1.5-fold change being considered statistically significant.

For RNA-Seq analysis, strains were grown in quadruplicate to mid-exponential phase and RNA was isolated as for TaqMan qRT-PCR. First and second strand cDNA synthesis was performed on 500 ng of RNA using the Ovation Prokaryotic RNA-Seq System (NuGEN). The resulting cDNA was fragmented to 200 bp (mean fragment size) with the S220 Focused-ultrasonicator (Covaris) and used to make barcoded sequencing libraries on the SPRI-TE Nucleic Acid Extractor (Beckman-Coulter). No size selection was employed. Libraries were quantitated by qPCR (KAPA Systems), multiplexed and 8 samples per lane were sequenced on the HiSeq2000 using 76 bp paired-end sequencing. The raw reads in FASTQ format were aligned to the reference genome, *Streptococcus pyogenes* MGAS315 (GI:21905618), using Mosaik alignment software (version: 1.1.0021, http://code.google.com/p/mosaik-aligner/) with the following alignment parameters: 8 percent maximal percentage read length allowed to be errors and 50 percent minimal percentage of the read length aligned. Duplicate fragments with a lower alignment quality were discarded. Next, the overlaps between aligned reads and annotated genes were counted using HTSeq software (http://www-huber.embl.de/users/anders/HTSeq/doc/overview.html). If the number of overlapped read of any given gene was less than one per million total mapped read for all samples, this gene was excluded from further analysis. 330 (17%) of total 1951 genes were removed due to low expression. The gene counts were normalized using the scaling factor method [78]. A negative binomial generalized linear model was fitted to each gene. Then a likelihood-ratio test was applied to examine if there was a difference in the gene transcript levels among the six strains [78]. The Benjamini-Hochberg method was used to control false discovery rate (FDR) [79]. Next, pair-wise comparisons using the likelihood ratio test were performed to compare the gene transcript levels between pairs of strains with the Holm's method used to calculate adjusted *P*-values to correct for multiple testing. Transcript levels were considered significantly different only if the mean transcript level difference was ≥1.5-fold and the final, adjusted *P* value was less than 0.05.

### Electrophoretic shift assays

The ∼300 bp encompassing promoter regions of selected CovR regulated genes were amplified by PCR from MGAS10870 genomic DNA using the respective primer pairs listed in Table S2. The purified PCR products (0.4 µg) were incubated with indicated amounts of various CovR isoforms at 37°C for 15 min in TBE-buffer (89 mM Tris, 89 mM borate, 1 mM EDTA, 5% glycerol, and 10 µg/ml polydI:dC (Sigma)) as described [43]. Samples were then separated on a 6% TBE-PAA gel for 70 min at 120 V and stained with ethidium bromide.

### Mouse infection studies/*in vivo* gene expression analysis

20 female outbred CD-1 Swiss mice per strain (Harlan-Sprague-Dawley) were injected intraperitoneally with 1.0×10^7^ GAS CFU and monitored for near-mortality. Differences in survival were calculated using a Kaplan-Meier survival analysis with Bonferroni's correction for multiple comparisons. For *in vivo* gene expression assays, four mice per strain were euthanized 24 hours after infection and blood was isolated via cardiac puncture. Total RNA was isolated from blood using the QIAamp RNA Blood Mini Kit (Qiagen) with DNA contamination being eliminated using Turbo DNAfree (Ambion). Bacterial RNA was preferentially isolated from the total blood RNA using the MICROBEnrich Kit (Invitrogen) according to the manufacturer's instruction. cDNA was created from the RNA as previously described. RNA samples without reverse transcriptase were included as negative controls to ensure that no contaminating DNA was present in any of the samples. TaqMan real-time qRT-PCR was performed in triplicate on an Applied Biosystems Step-One Plus system as previously described [77].

### Accession numbers

RNA-Seq data have been deposited at the short-read archive under accession # PRJNA238945.

## Supporting Information

Text S1 **Supporting figures.** This document contains Figures S1–S5.
(DOCX)

Table S1 **Primers and probes used in this study.**
(DOC)

Table S2 **Summary of RNA-seq data.**
(XLSX)
