# Supplementary material for: Dual-Site Phosphorylation of the Control of Virulence Regulator Impacts Group A Streptococcal Global Gene Expression and Pathogenesis
Source: PLoS Pathog. 2014 May 1;10(5):e1004088. doi: 10.1371/journal.ppat.1004088 (PMC4006921; doi:10.1371/journal.ppat.1004088)
Supplement: Text S1 — Supporting figures. This document contains Figures S1–S5. (DOCX) [file ppat.1004088.s001.docx]

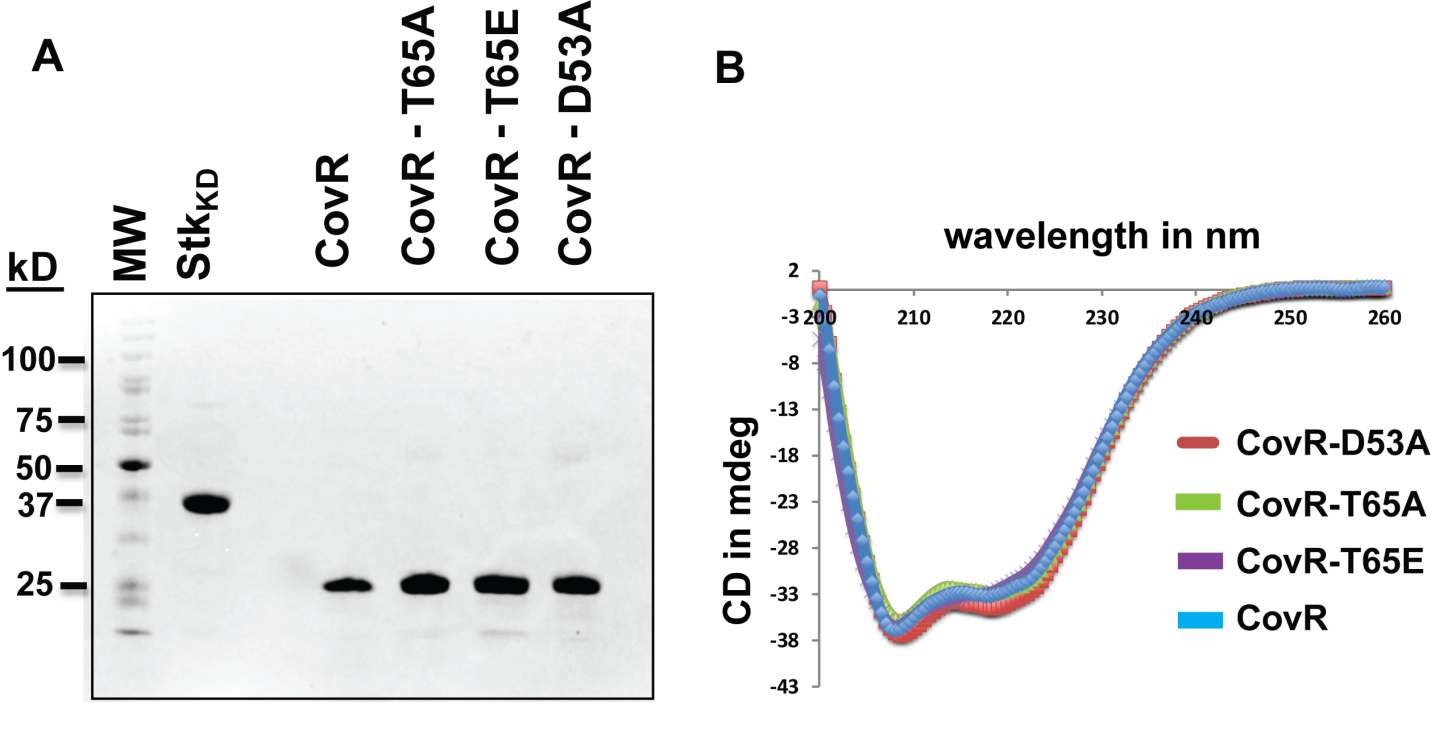


**Figure S1 in Text S1.** Analysis of recombinant proteins. (A) SDS-PAGE of indicated recombinant proteins run on a 12% gel. MW = molecular weight ladder. Stk_KD_ = kinase domain of Stk. (B) Circular dichromism assay demonstrating the similar folding characteristics of the various recombinant CovR proteins. Far-UV (200-260nm) CD spectra of CovR wild type (blue), CovR-T65E (purple), CovR-T65A (green) and CovR-D53A (red).





**Figure S2 in Text S1.** Performance of anti-CovR antibody. MGAS10870 was grown to mid-exponential phase and indicated amount of cell lysates were run on a 12% SDS polyacrylamide gel, transferred to a nitrocellulose membrane, and probed using an anti-CovR antibody. The numbers represent μg total protein loaded on the gel, respectively. Indicated amounts of recombinant CovR were included in the first and last lanes.


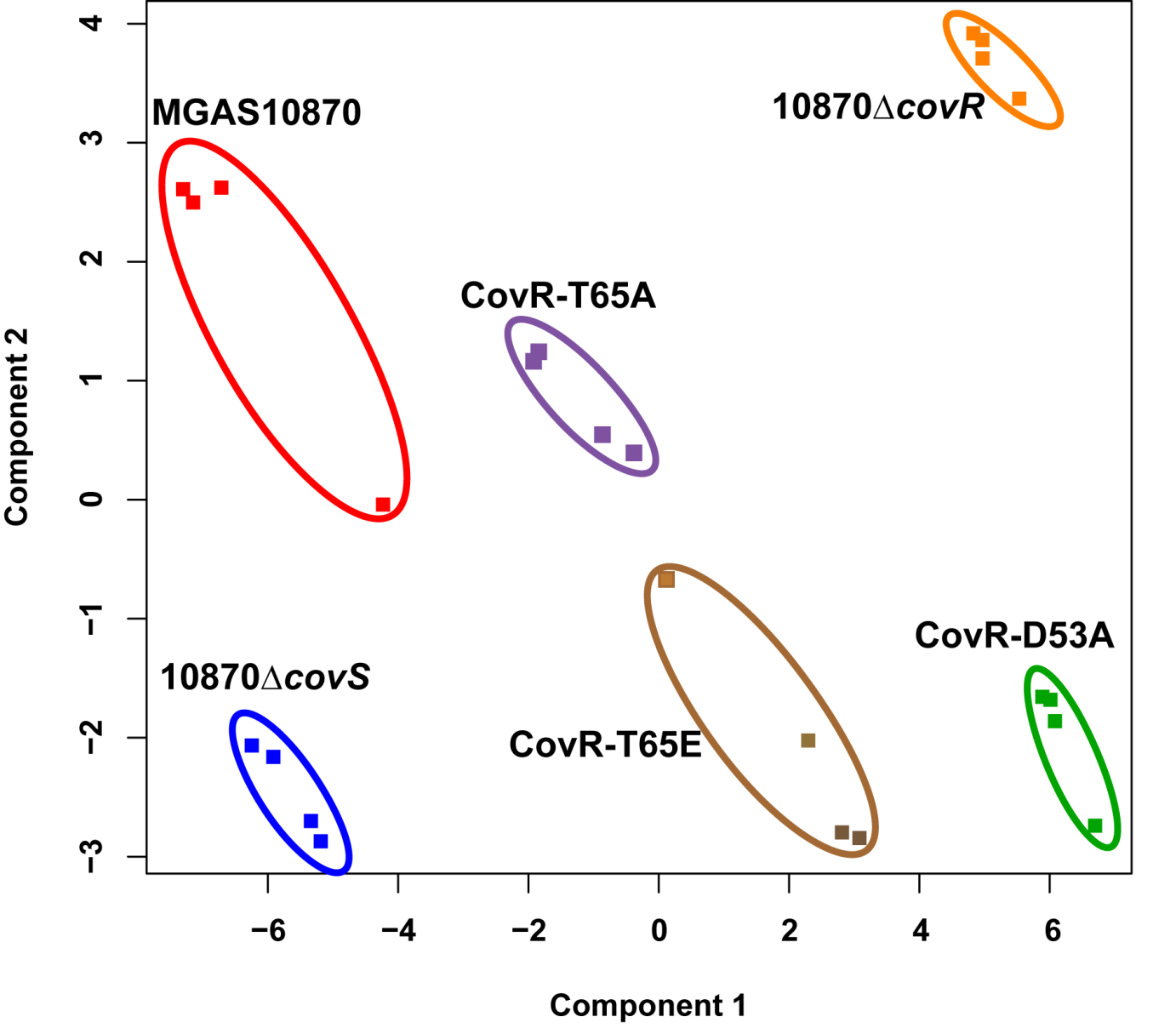


**Figure S3 in Text S1.** Principal components analysis (PCA) showing inter-sample variation for any differentially expressed transcripts detected in pairwise comparisons to wild type strain (fold change > 1.5 and adjusted *P* value < 0.05). The majority of the variation (61%) is depicted by location along the horizontal axis.


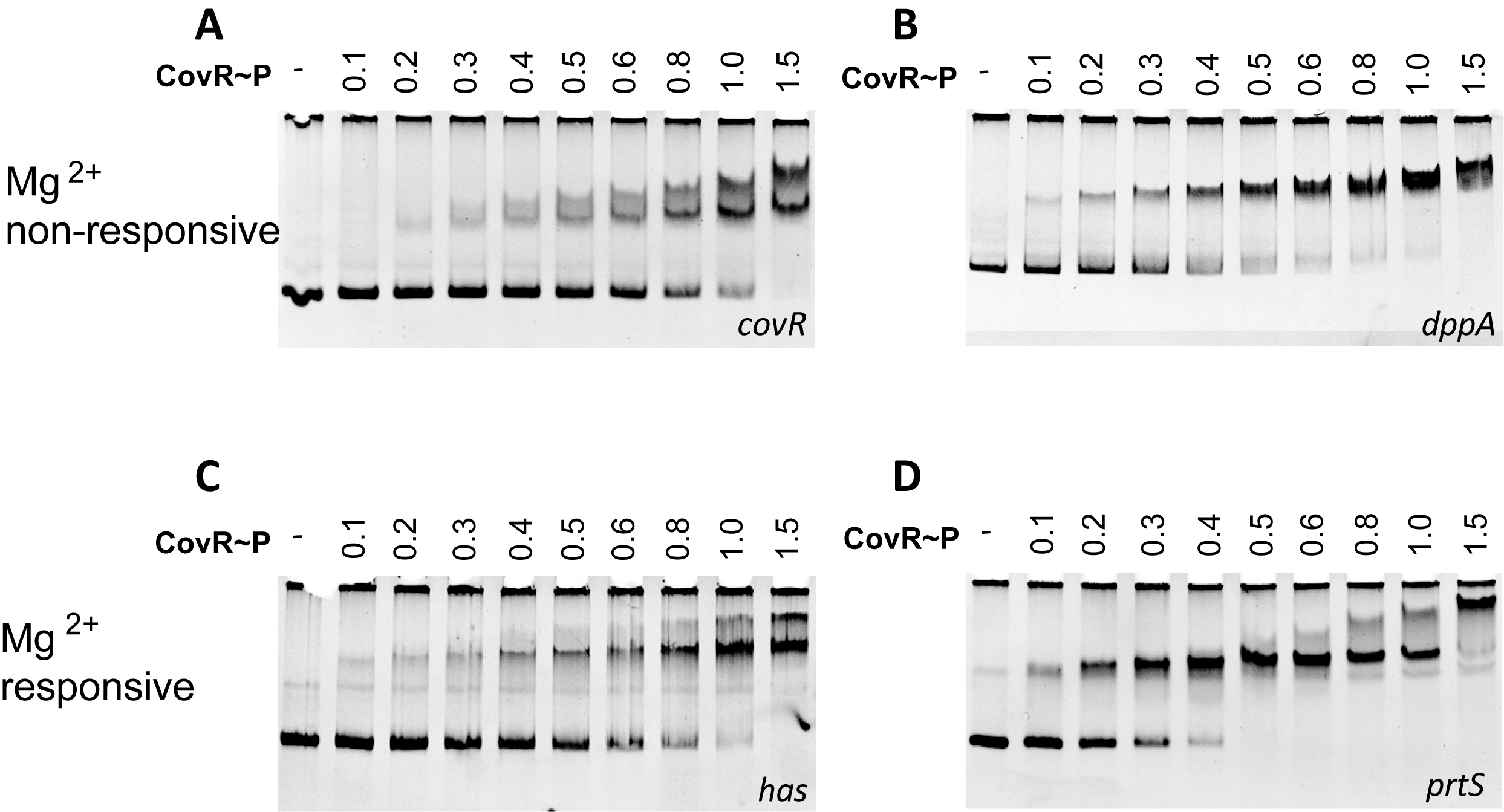


**Figure S4 in Text S1. Responsiveness to Mg^2+^ does not correspond with reduced DNA binding affinity of CovR-D53~P.** Electromobility shift assays (EMSA) analyzing the affinity of recombinant CovR-D53~P to the Mg^2+^-non-responsive promoters of *covR* (*A*) and *dppA* (B) and the Mg^2+^-responsive promoters *hasA* (C) and *prtS* (D). Increasing concentrations of CovR (monomer concentration given in μM) phosphorylated *in vitro* using acetyl-phosphate were used as indicated. Samples were incubated for 15 min at 37 °C and electrophoresed on a 6% TBE polyacrylamide gel for 60 min at 120 V. The gels were stained with ethidium bromide. ns, non-specific DNA, f, free DNA, cI, lower molecular weight complex, cII, higher molecular weight complex. Gels shown are representatives of identical results obtained on two separate occasions.


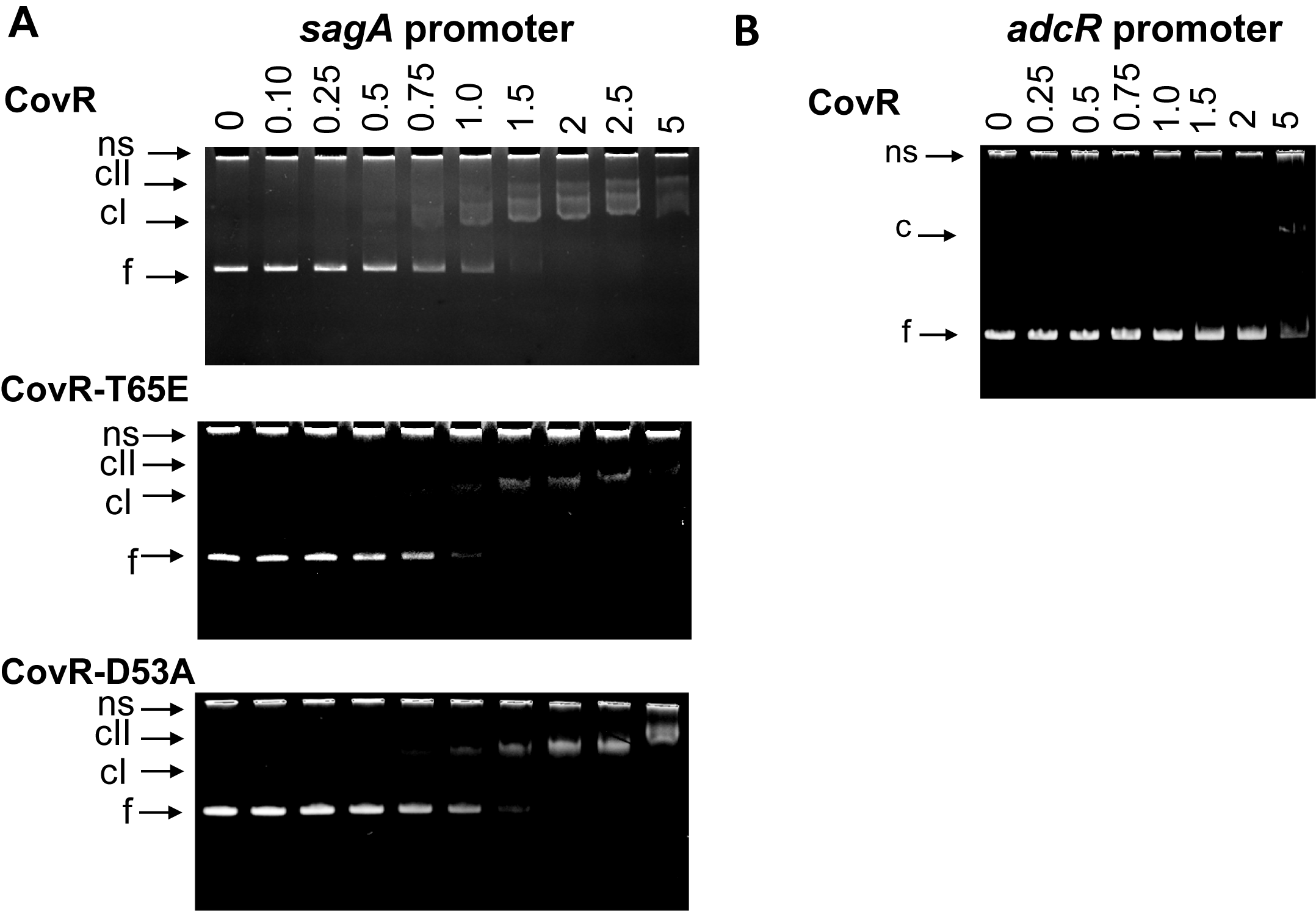


**Figure S5 in Text S1. CovR-D53A and CovR-T65E bind CovR-regulated promoters with similar affinity to unphosphorylated CovR.** (A) Electrophomobility shift assays (EMSA) measuring binding of recombinant CovR, CovR-D53A, and CovR-T65E proteins to the promoter of *sagA*. Increasing concentrations of unphosphorylated CovR, CovR-D53A, and CovR-T65E (monomer concentration given in μM) were used as indicated. Samples were incubated for 15 min at 37 °C and electrophoresed on a 6% TBE polyacrylamide gel for 60 min at 120 V. The gels were stained with ethidium bromide. ns, non-specific DNA, f, free DNA, cI, lower molecular weight complex, cII, higher molecular weight complex. (B) Binding of recombinant CovR to the promoter of *adcR*, which is not regulated by CovR is shown to illustrate specificity of CovR binding. For both (A) and (B) gels shown are representatives of identical results obtained on two separate occasions.
