# Supplementary material for: Dual-Site Phosphorylation of the Control of Virulence Regulator Impacts Group A Streptococcal Global Gene Expression and Pathogenesis
Source: PLoS Pathog. 2014 May 1;10(5):e1004088. doi: 10.1371/journal.ppat.1004088 (PMC4006921; doi:10.1371/journal.ppat.1004088)
Supplement: Table S1 — Primers and probes used in this study. (DOC) [file ppat.1004088.s002.doc]

**Table S1. Primers and probes used in this study.**

| Primer | Sequence (5’→3’) | Target |
| --- | --- | --- |
| covS-A | AGA GGG CAG AGA AGG TAA TGA CTA | 5’ primer for amplifying *covS* gene region |
| covS-B | GTT ATA GTT ATT ATA ACA TGT ATT GGC AAT CAG TGT AAA GGC AGA GA | 3’ primer for amplifying *covS* gene region with *spc* cassette overlap |
| covS-C | CTA TTT AAA TAA CAG ATT AAA AAA ATT ATA ATG GCC CAG TCT AAA GAG AGT TAG AG | 5’ primer for amplifying *covS* gene region with spc cassette overlap |
| covS-D | AGG CTG CGT CAC TTC CAA TAA T | 3’ primer for amplifying *covS* gene region |
| covS-spcF | TCT CTG CCT TTA CAC TGA TTG CCA ATA CAT GTT ATA ATA ACT ATA AC | 5’ primer for amplifying spc cassette with GAS *covS* overlap |
| covS-spcR | CTC TAA CTC TCT TTA GAC TGG GCC ATT ATA ATT TTT TTA ATC TGT TAT TTA AAT AG | 3’ primer for amplifying spc cassette with GAS *covS* overlap |
| covR-pJL 5’ | AGT CGG ATC CTA ATC CTT TTG CTA GCT TG | 5’ primer for amplifying *covR* gene region for cloning into pJL |
| covR-pJL 3’ | CAG TCT CGA GCC AGG AGA TGA TTC TTC GTT AT | 3’ primer for amplifying *covR* gene region for cloning into pJL |
| covR-D53A-5’ | GAT TTA ATC CTG CTT GCC TTA ATG TTA CCA GAG | 5’ primer for site changing CovR amino acid 53 from aspartate to alanine |
| covR-D53A-3’ | CTC TGG TAA CAT TAA GGC AAG CAG GAT TAA ATC | 3’ primer for site changing CovR amino acid 53 from aspartate to alanine |
| covR-T65A-5’ | GAT GGT TTT GAA GTG GCC cgt cgt ttg caa acc | 5’ primer for site changing CovR amino acid 65 from threonine to alanine |
| covR-T65A-3’ | GGT TTG CAA ACG ACG GGC CAC TTC AAA ACC ATC | 5’ primer for site changing CovR amino acid 65 from threonine to alanine |
| covR-T65E-5’ | GAT GGT TTT GAA GTG GAA cgt cgt ttg caa acc | 5’ primer for site directed mutagenesis of codon encoding CovR amino acid 193 |
| covR-T65E-3’ | GGT TTG CAA ACG ACG TTC CAC TTC AAA ACC ATC | 3’ primer for site directed mutagenesis of codon encoding CovR amino acid 193 |
| Stk1_fwd: | ATA ATA ATA CAT ATG ATT CAG ATT GGC AAA TTA TTT G | 5’ primer for cloning Stk kinase domain |
| Stk1_rev: | ATA ATA ATA CTC GAG TTA TAA CAC TGT TGG GGT AGG | 3’ primer for cloning Stk kinase domain |
| *ska-*prom-5’ | CAT TTT CTG ACT TTT TTA ATC ATT | 5’ primer for amplifying *ska* promoter |
| *ska*-prom-3’ | GTT AAG TTT CAA TCC CCA TTA | 3’ primer for amplifying *ska* promoter |
| *sagA-*prom-5’ | GAT TCG CTT AAT AAA GAC ATT TC | 5’ primer for amplifying *sagA* promoter |
| *sagA*-prom-3’ | GAA GTA AAT TTT AAC ATA AGG T | 3’ primer for amplifying *sagA* promoter |
| *hasA-*prom-5’ | CCT TTA CCA GTT ATC ATA TTT C | 5’ primer for amplifying *hasA* promoter |
| *hasA*-prom-3’ | GAT AGA TAT CAA AAA AAT AAA GG | 3’ primer for amplifying *hasA* promoter |
| 5' *covRS* PCR | GAC CAT AGA GGG CAG AGA AGG | 5’ primer for PCR for *covRS* operon sequencing |
| 3’ *covRS* PCR | CAG TTG TGG CAA AAG ACT GC | 3’ primer for PCR for *covRS* operon sequencing |
| *covRS* seq1 | AAA ATT TGT GGG CTA TGT TCA AGT | *covRS* sequencing primer |
| *covRS* seq2 | GTC AGA AAA TCC AAA AAC GAT AGC | *covRS* sequencing primer |
| *covRS* seq3 | AAT ATC ACT GTC ATC TTT ACC CCA A | *covRS* sequencing primer |
| *covRS* seq4 | TAG AGT ATT TGT GAT ATC GCG CTC | *covRS* sequencing primer |
| *ska* QRT 5’ | GCT GAC AAA GAT GGT TCG GTA AC | 5’ primer for *ska* QRT-PCR |
| *ska* QRT 3’ | CAC ATG CCC GCT TAG CAA A | 3’ primer for *ska* QRT-PCR |
| *ska* QRT probe | TTG CCG ACC CAA CCT GTC CAA GAA | Probe for *ska* QRT-PCR |
| *prtS* QRT 5’ | AAG GAG CTT GGG ACA AGG GAT A | 5’ primer for *prtS* QRT-PCR |
| *prtS* QRT 3’ | TGA TGG GCC GGA TCG A | 3’ primer for *prtS* QRT-PCR |
| *prtS* probe | AAG GAG CTT GGG ACA AGG GAT A | Probe for *prtS* QRT-PCR |
| *sagA* QRT 5’ | TTG CTC CTG GAG GCT GCT | 5’ primer for *sagA* QRT-PCR |
| *sagA* QRT 3’ | CTT CCG CTA CCA CCT TGA GAA T | 3’ primer for *sagA* QRT-PCR |
| *sagA* probe | ACC ACT TCC AGT AGC AAT TGA GAA GCA ACA AG | Probe for *sagA* QRT-PCR |
| *hasA* QRT 5’ | ACC GTT CCC TTG TCA ATA AAG G | 5’ primer for *hasA* QRT-PCR |
| *hasA* QRT 3’ | CGT CAG CGT CAG ATC TTT CAA A | 3’ primer for *hasA* QRT-PCR |
| *hasA* probe | CGC CAT GCT CAA GCG TGG GC | Probe for *hasA* QRT-PCR |
| *covR* QRT 5’ | GGT GCA GAC GAC TAT ATT GTT AAA CCC | 5’ primer for *covR* QRT-PCR |
| *covR* QRT 3’ | TTG ACG GCG GAA AAT AGC A | 3’ primer for *covR* QRT-PCR |
| *covR* probe | TTT GCC ATT GAA GAA CTA CTT GCC CGT ATT C | Probe for *covR* QRT-PCR |
| *dppA* QRT 5’ | TTT ATG CCG GAG GAC GTC AT | 5’ primer for *dppA* QRT-PCR |
| *dppA* QRT 3’ | CTT TCC CTG CTA GGC TTG GA | 3’ primer for *dppA* QRT-PCR |
| *dppA* probe | CGC GCA GCA ATT TTA TGA ATC GCA T | Probe for *dppA* QRT-PCR |
| *mac-1* QRT 5’ | ATC GAA CCA CCA GTG AAG CAT | 5’ primer for *mac-1* QRT-PCR |
| *mac-1* QRT 3’ | ACA TTC AAT GGA AAA GAC GAT CTT CT | 3’ primer for *mac-1* QRT-PCR |
| *mac-1* probe | TTC CCT GGT GTG GCA GCC CC | Probe for *mac-1* QRT-PCR |
| *speB* QRT 5’ | CGC ACT AAA CCC TTC AGC TCT T | 5’ primer for *speB* QRT-PCR |
| *speB* QRT 3’ | ACA GCA CTT TGG TAA CCG TTG A | 3’ primer for *speB* QRT-PCR |
| *speB* probe | GCC TGC GCC GCC ACC AGT A | Probe for *speB* QRT-PCR |
| *spyM3_0105* QRT 5’ | TTA CAA ACT AAA AAA CGA CGG GAA A | 5’ primer for *spyM3_0105* QRT-PCR |
| *spyM3_0105* QRT 3’ | ATT CCA TTT ATC TTT GTA ACC TTC TTC AAG | 3’ primer for *spyM3_0105* QRT-PCR |
| *spyM3_0105* probe | AGA CAC CTA CGG CCG TCA ATC ACA CAC | Probe for *spyM3_0105* QRT-PCR |
| *spyM3_0132* QRT 5’ | TGA TCG CAA CTG CTG CTG TT | 5’ primer for *spyM3_0132* QRT-PCR |
| *spyM3_0132* QRT 3’ | AGG ACC AGT TGT GTC CGT AGA AAC | 3’ primer for *spyM3_0132* QRT-PCR |
| *spyM3_0132* probe | AGC CTT GCA ACA CTA GGA GAC CAC AAA A | Probe for *spyM3_0132* QRT-PCR |
| *spyM3_1493* QRT 5’ | AGC TCA TGC CCT ACA CAA TGA A | 5’ primer for *spyM3_1493* QRT-PCR |
| *spyM3_1493* QRT 3’ | AAT CTC TGG GTG TGA CAT AGT AAC ATC T | 3’ primer for *spyM3_1493* QRT-PCR |
| *spyM3_1493* probe | CAG TCC CTC AAC CAA AGC AGC TCG TT | Probe for *spyM3_1493* QRT-PCR |
| *tufA* QRT 5’ | CAA CTC GTC ACT ATG CGC ACA T | 5’ primer for *tufA* QRT-PCR |
| *tufA* QRT 3’ | GAG CGG CAC CAG TGA TCA T | 3’ primer for *tufA* QRT-PCR |
| *tufA* probe | CTC CAG GAC ACG CGG ACT ACG TTA AAA A | Probe for *tufA* QRT-PCR |
